# Placental RTN3L‐dependent ER‐Phagy Contributes to Fetal Testicular Dysplasia Upon Environmental Stress

**DOI:** 10.1002/advs.202500924

**Published:** 2025-04-26

**Authors:** Ye‐Xin Luo, Hua‐Long Zhu, Bin‐Bin Huang, Cheng‐Fang Sun, Xin‐Xin Zhang, Xin‐Run Wang, Yi‐Fan Hu, Xu‐Dong Zhang, Shen‐Dong Xu, Huan Zhou, Rui Pan, Wei Chang, Zhi Yuan, Yong‐Wei Xiong, Xiao‐Feng Xu, Ling‐Li Zhao, De‐Xiang Xu, Hua Wang

**Affiliations:** ^1^ Department of Toxicology Center for Big Data and Population Health of IHM School of Public Health Anhui Medical University Hefei 230022 China; ^2^ Key Laboratory of Environmental Toxicology of Anhui Higher Education Institutes Hefei 230022 China; ^3^ Key Laboratory of Population Health Across Life Cycle (Anhui Medical University) Ministry of Education of The People's Republic of China Hefei 230022 China; ^4^ Department of Prenatal Diagnosis Wuxi Maternity and Child Care Hospital Wuxi Jiangsu 214000 China; ^5^ Department of Maternal Child & Adolescent Health School of Public Health Anhui Medical University Hefei 230022 China; ^6^ Reproductive Medicine Center Department of Obstetrics and Gynecology the First Affiliated Hospital of Anhui Medical University Hefei 230022 China

**Keywords:** environmental stress, ER‐phagy, fetal testis development, m6A, placental estradiol

## Abstract

Prenatal environmental stress damages fetal testicular development, leading to male infertility. However, the precise mechanisms underlying the impact of gestational environmental stress on fetal testicular development require further investigation. This study demonstrates that gestational environmental stressor cadmium exposure caused placental estradiol synthesis inhibition and fetal testicular dysplasia. Gestational estradiol supplementation restores fetal testicular dysplasia caused by environmental stress‐induced placental estradiol synthesis inhibition. Analysis of human placentae and cadmium‐stimulated human primary placental trophoblasts confirmed that ER‐phagy is associated with the inhibition of estradiol synthesis in placentae. Subsequently, the data reveals that environmental stress significantly activates RTN3L‐mediated ER‐phagy. *RTN3L*‐deficient cells and placental *Rtn3l*‐specific knockout mice confirm that environmental stress‐activated RTN3L‐mediated ER‐phagy inhibited placental estradiol synthesis. Total N6‐methyladenosine level increasing in gestational environmental stress‐exposed placentae. METTL3‐mediated N6‐methyladenosine modification suppression obviously restrains environmental stress‐activated RTN3L‐dependent ER‐phagy. In conclusion, gestational environmental stress activates ER‐phagy by increasing placental *Rtn3l* mRNA N6‐methyladenosine modification, inhibiting placental estradiol synthesis, and contributing to fetal testicular dysplasia. The study demonstrates the early prevention and treatment of adult male infertility from the perspective of fetal‐derived diseases.

## Graphical abstract

Gestational environmental stress activates ER‐phagy by increasing placental *Rtn3l* mRNA N6‐methyladenosine modification, further inhibiting placental estradiol synthesis and contributing to fetal testicular dysplasia. This study demonstrates the early prevention and treatment of adult male infertility from the perspective of fetal‐derived diseases.

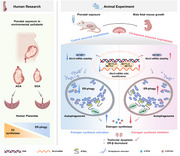

## 1 Introduction

About 15% of couples worldwide are infertile, and male factors are estimated to be responsible for 30–50% of infertility cases.^[^ ^1^ ^]^ Fetal testicular development is related to adult testicular function, and fetal testicular dysplasia increases the probability of adult male infertility.^[^ ^2^, ^3^, ^4^, ^5^ ^]^ Gestational exposure to environmental stressors, such as perfluorooctanoic sulfonate and bisphenol A, impairs fetal testicular development and further affects male fertility in offspring.^[^ ^6^, ^7^ ^]^ Cadmium (Cd) is a classical environmental stressor with reproductive and developmental toxicity.^[^ ^8^, ^9^ ^]^ The previous population study reported a negative correlation between maternal Cd exposure and fetal testicular development.^[^ ^10^ ^]^ Several animal studies have shown that maternal Cd exposure during pregnancy affects the development and function of the fetal testis.^[^ ^11^, ^12^ ^]^ However, the mechanism by which environmental stress impairs fetal testicular development remains unclear. β‐estradiol (E2), the main form of estrogen, has been reported to promote fetal testis development and male fertility.^[^ ^13^, ^14^, ^15^, ^16^, ^17^ ^]^ Moreover, a population study has reported that environmental stress is negatively correlated with E2 levels in cord blood and fetal growth.^[^ ^18^ ^]^ Other animal experiments have shown that maternal environmental stress impairs fetal muscle and nerve development by decreasing estrogen levels.^[^ ^19^, ^20^ ^]^ Maternal environmental stress exposure may affect fetal testicular development by reducing estrogen levels. Most of the E2 during pregnancy comes from the maternal ovaries and placentae and then is passed from the placenta to the fetus.^[^ ^21^, ^22^ ^]^ Notably, E2 mainly comes from the maternal ovary in early pregnancy, whereas the placentae replace the ovaries as the main organ of E2 synthesis after the 8th week of gestation.^[^ ^23^, ^24^ ^]^ Our previous studies also indicated that gestational environmental stress exposure blocked E2/ER signaling in the fetal liver via repressing placental E2 synthesis.^[^ ^25^ ^]^ Therefore, we hypothesize that environmental stress impairs fetal testicular development by reducing placenta‐derived E2 synthesis. However, the underlying mechanism by which maternal Cd exposure inhibits placental E2 synthesis remains unclear.

Endoplasmic reticulum autophagy (ER‐phagy) is a process in which damaged endoplasmic reticulum (ER) is selectively degraded via lysosomes in eukaryotic cells.^[^ ^26^ ^]^ Under physiological conditions, ER‐phagy contributes to maintaining ER protein homeostasis, thereby preserving cell function and survival.^[^ ^27^ ^]^ However, excessive ER‐phagy promotes ER protein loss, leading to cell dysfunction and death.^[^ ^28^ ^]^ Previous studies have primarily focused on the role of ER‐phagy in kidney injury and neurological dysfunction.^[^ ^29^, ^30^ ^]^ However, no studies have hitherto focused on the role of ER‐phagy in the impairment of the pregnancy tissue placenta. CYP17A1 and CYP19, two ER proteins, are key rate‐limiting enzymes that catalyze E2 synthesis. In the ER, CYP17A1 converts progesterone to androstenedione, and then CYP19 converts androstenedione to estradiol.^[^ ^31^ ^]^ Previous studies have found that environmental stressor Cd exposure during pregnancy reduced the level of placental E2 synthase, such as CYP19.^[^ ^32^ ^]^ A recent study shows that the environmental stressor ochratoxin A activates excessive ER‐phagy, inducing ER protein loss in human proximal tubule epithelial cells.^[^ ^28^ ^]^ Therefore, we hypothesize that maternal environmental stress during pregnancy could inhibit placental E2 synthesis via triggering ER‐phagy.

N6‐methyladenosine (m6A) is a common eukaryotic mRNA modification where a methyl group is added to the N6 position of adenosine in RNA molecules.^[^ ^33^ ^]^ m6A modification is regulated by a series of writers (METTL3 and METTL14), erasers (ALKBH5 and FTO), and readers (IGF2BPs, YTHDFs, and YTHDCs).^[^ ^34^ ^]^ m6A modification is involved in the regulation of various cellular processes, including proliferation, differentiation, and cell death.^[^ ^35^ ^]^ In physiological conditions, m6A enhances the immune tolerance of placental trophoblast cells, promotes the stability of placental function, and maintains normal pregnancy.^[^ ^36^ ^]^ Pathologically, m6A modification inhibits the proliferation of human trophoblast cells and induces abortion.^[^ ^37^ ^]^ Our recent study showed that environmental stress increased placental m6A modification levels to impair fetal development.^[^ ^38^ ^]^ Previous studies further indicated that m6A modification promoted the activation of non‐selective autophagy in bone marrow mesenchymal stem cells, hepatic cells, and mouse embryonic fibroblasts.^[^ ^39^, ^40^, ^41^ ^]^ However, the role of m6A in the activation of placental ER‐phagy, selective autophagy, remains to be clarified. ER‐phagy selectivity relies on receptors like RTN3L, FAM134B, and SEC62, which specifically target ERs to autophagosomes.^[^ ^42^ ^]^ Another recent study reported that m6A modification activated mitophagy by enhancing mRNA stability of the mitophagy receptor *Bnip3* in cardiomyocytes.^[^ ^43^ ^]^ As mentioned above, we hypothesize that gestational environmental stress exposure increases m6A levels of ER‐phagy receptors mRNA and activates placental ER‐phagy.

In this study, we established an animal model of fetal testicular development impaired by environmental stress during pregnancy. Exogenous E2 supplementation and ovariectomy were used to explore the role of reduced placental‐derived E2 in environmental stress‐impaired fetal testicular development. *RTN3L‐*deficient JEG‐3 cells and placental *Rtn3l‐*specific knockout mice were generated to clarify the role of activated ER‐phagy in environmental stress‐inhibited placental E2 synthesis. Supplementation with S‐adenosylhomocysteine and STM2457, two METTL3 inhibitors, were performed to investigate the role of elevated m6A modification in placental ER‐phagy activation induced by environmental stress. These findings will inspire earlier prevention and treatment options for adult male infertility from its fetus‐derived disease perspective.

## 2 Results

### 2.1 Environmental Stress Impairs Fetal Growth and Testicular Development via Inhibiting Estradiol Signaling in Mice

**Figure 1:**
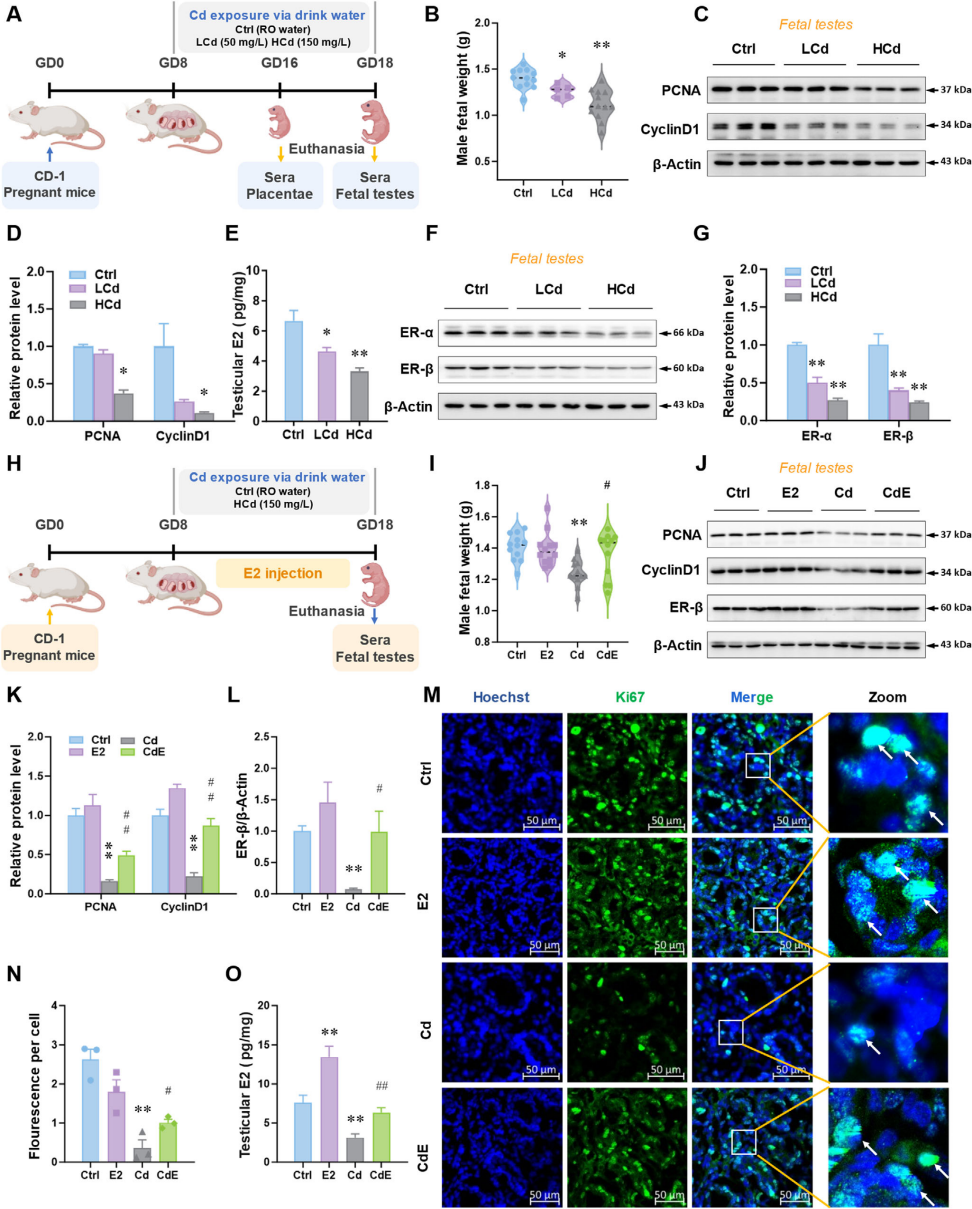
Environmental stress impairs fetal growth and testicular development via inhibiting estradiol signaling in mice. A–G) The pregnant mice were exposed to low concentrations of CdCl_2_ (50 mg L^−1^, LCd) or high concentrations of CdCl_2_ (150 mg L^−1^, HCd) by drinking water from gestational day (GD) 8 to GD18. After euthanasia, sera, placentae, and fetal testes were collected on GD18. A) Animal experiment diagram. B) Male fetal weight. C,) Protein levels of PCNA and CyclinD1 in fetal testes. E) Estradiol levels in fetal testes. Estradiol levels are pico per milligram of testes. F,G) Protein levels of ERα and ERβ in fetal testes. H–O) The pregnant mice were exposed to high concentrations of CdCl_2_ (150 mg L^−1^, Cd) by drinking water paralleled with estradiol intervention from GD8 to GD18. After euthanasia, sera, placentae, and fetal testes were collected on GD18. H) Animal experimental diagram of estradiol intervention. I) Weight of male fetal mice. J–L) Protein levels of PCNA, CyclinD1, and ERβ in fetal testes. M,N) Ki67 immunostaining of fetal testes. Hoechst33258 was used to tag the nucleus. O) Estradiol levels in fetal testes. Estradiol levels are pico per milligram of testes. All data were analyzed using one‐way *ANOVA* and presented as means *± SEMs*. *n* = 3–12. ^*^ *p*<0.05, ^**^ *p*<0.01, compared to Ctrl; ^#^ *p*<0.05, ^##^ *p*<0.01, compared to Cd.

To investigate the effect of gestational environmental stress on fetal growth and testicular development, pregnant mice were exposed to cadmium (Cd), a typical environmental stressor (**Figure** **1**A). We found that Cd caused fetal growth restriction in male mice (Figure 1B). The expression of PCNA and CyclinD1, two proteins associated with proliferation, was also downregulated in fetal testes (Figure 1C,D). Immunofluorescent analysis indicated that the number of Ki67^+^ cells was reduced in fetal testes in response to environmental stress (Figure S1A,B, Supporting Information). Furthermore, E2 levels were significantly reduced in fetal testes upon environmental Cd exposure (Figure 1E). Meanwhile, the expression of ER‐α and ER‐β, two E2 receptors, was also downregulated in fetal testes (Figure 1F,G). To explore the effect of reduced E2 signaling in environmental stress‐inhibited testicular development, pregnant mice were exposed to Cd with estradiol supplementation (Figure 1H). The results displayed that E2 supplementation reversed the decrease in male fetal weight induced by prenatal stress (Figure 1I). Compared to Cd group, the levels of PCNA, CyclinD1, and ER‐β proteins in CdE (Cd + Estradiol) group were higher in fetal testes (Figure 1J–L). As expected, the number of Ki67^+^ cells in fetal testes in CdE group was greater than that in Cd group (Figure 1M,N). E2 supplementation also recovered Cd‐induced reduction of E2 levels in fetal testes (Figure 1O). In summary, gestational environmental stress impairs fetal testicular development via blocking E2 signaling.

### 2.2 Placental Estradiol Synthesis Inhibition Mediates Fetal Testicular Estradiol Signaling Repression Caused via Environmental Stress

**Figure 2:**
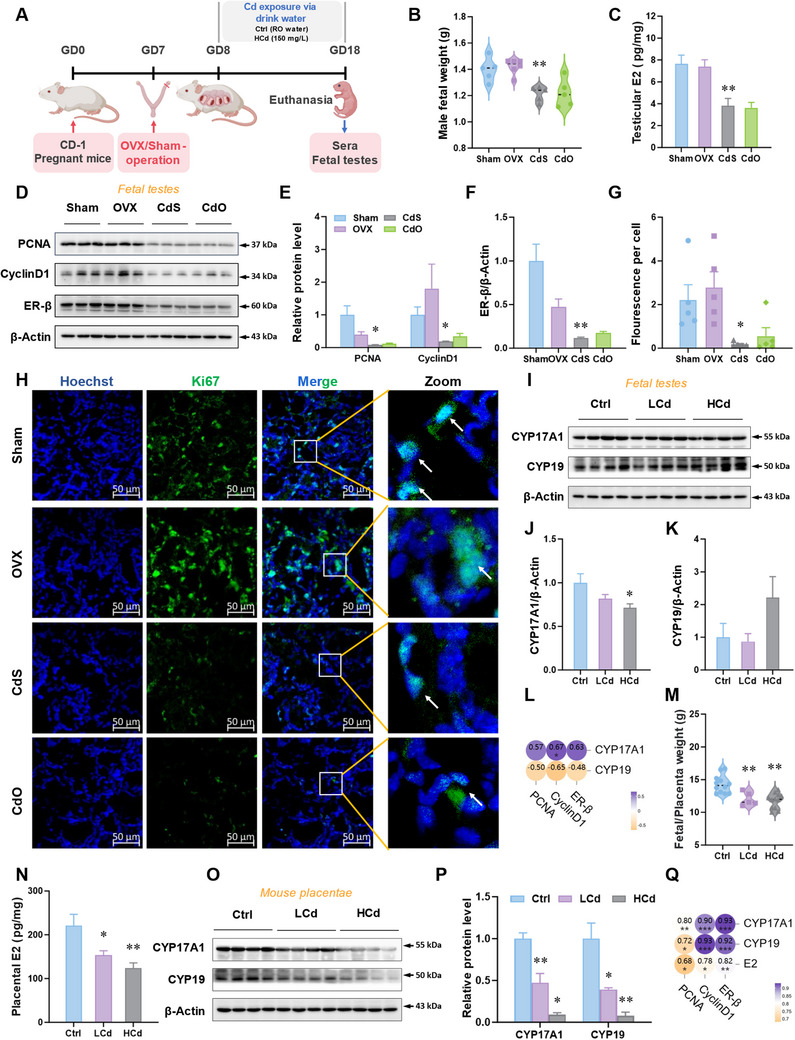
Placental estradiol synthesis inhibition mediates fetal testicular estradiol signaling repression caused via environmental stress. A–H) The pregnant mice were ovariectomized on gestational day (GD) 7. Then, the pregnant mice were exposed to high concentrations of CdCl_2_ (150 mg L^−1^, Cd) by drinking water from GD8 to GD18. After euthanasia, sera and fetal testes were collected on GD18. A) Animal experiment diagram of ovariectomy. B) Male fetal weight. C) Estradiol levels in fetal testes. Estradiol levels are pico per milligram of testes. D–F) Protein levels of PCNA, CyclinD1, and ERβ in fetal testes. G,H) Ki67 immunofluorescent analysis of fetal testes. Hoechst33258 was used to tag the nucleus. I–Q) The pregnant mice were exposed to low concentrations of CdCl_2_ (50 mg L^−1^, LCd) or high concentrations of CdCl_2_ (150 mg L^−1^, HCd) by drinking water from GD8 to GD16. After euthanasia, sera, placentae, and fetal testes were collected on GD16. I–K) Protein levels of CYP17A1 and CYP19 in fetal testes. L) Visual representation of Spearman correlations among various indices and correlation strength indicated by the color depth. ^*^ *p*<0.05. M) The ratio of fetal weight to placental weight. N) Estradiol levels in mouse placentae. Estradiol levels are pico per milligram of placentae. O,P) Protein levels of CYP17A1 and CYP19 in mouse placentae. Q) Visual representation of Spearman correlations among various indices and correlation strength indicated by the color depth. ^*^ *p*<0.05, ^**^ *p*<0.01, ^***^ *p*<0.001. All data were analyzed using one‐way *ANOVA* and presented as mean *± SEMs*. *n* = 3–12. ^*^ *p*<0.05, ^**^ *p*<0.01, compared to Sham or Ctrl.

The sources of E2 during pregnancy can be divided into the maternal ovary, placenta, and fetus itself. To explore the mechanism by which environmental stress represses E2 signaling in fetal testes, ovariectomy or sham operation was performed on pregnant mice before Cd exposure (**Figure** **2**A). The data indicated that male fetal weight in CdO (Cd + OVX) group was similar to CdS (Cd + Sham) group (Figure 2B). Compared to CdS group, E2 levels in CdO group had no significant difference (Figure 2C). The expression of PCNA, CyclinD1, and ER‐β in CdO group was comparable to CdS group (Figure 2D–F). The number of Ki67^+^ cells in fetal testes in CdO group and CdS group were identical (Figure 2G,H). Accordingly, these data excluded the effect of ovariogenic E2. Furthermore, the expression of CYP17A1 and CYP19 in Cd‐exposed fetal testes, two proteins associated with E2 synthetase, was also equal to that in Ctrl group (Figure 2I–K). The above‐mentioned data and Spearman correlation analysis suggested that the effect of fetal testicular E2 synthesis was eliminated (Figure 2L). Moreover, gestational Cd exposure lowered the ratios of fetal weight to placental weight (Figure 2M). Further data manifested that placental E2 levels were reduced after Cd exposure (Figure 2N). RT‐qPCR results showed that Cd exposure during pregnancy did not change mRNA levels of estrogen synthetases *Cyp17a1* and *Cyp19* (Figure S2A, Supporting Information). However, the expression of CYP17A1 and CYP19 was also downregulated in placentae (Figure 2O,P). The above‐mentioned data and Spearman correlation analysis suggested that Cd‐exposed placental E2 synthesis inhibition impaired fetal testicular development (Figure 2Q). Similarly, Cd exposure also inhibited E2 synthesis in human JEG3 cells (Figure S2B,C, Supporting Information). As above, environmental stress represses E2 signaling in fetal testes via inhibiting placental E2 synthesis.

### 2.3 RTN3L‐Dependent ER‐Phagy Activated by Environmental Stress Inhibits Estradiol Synthesis in Mouse Placentae

**Figure 3:**
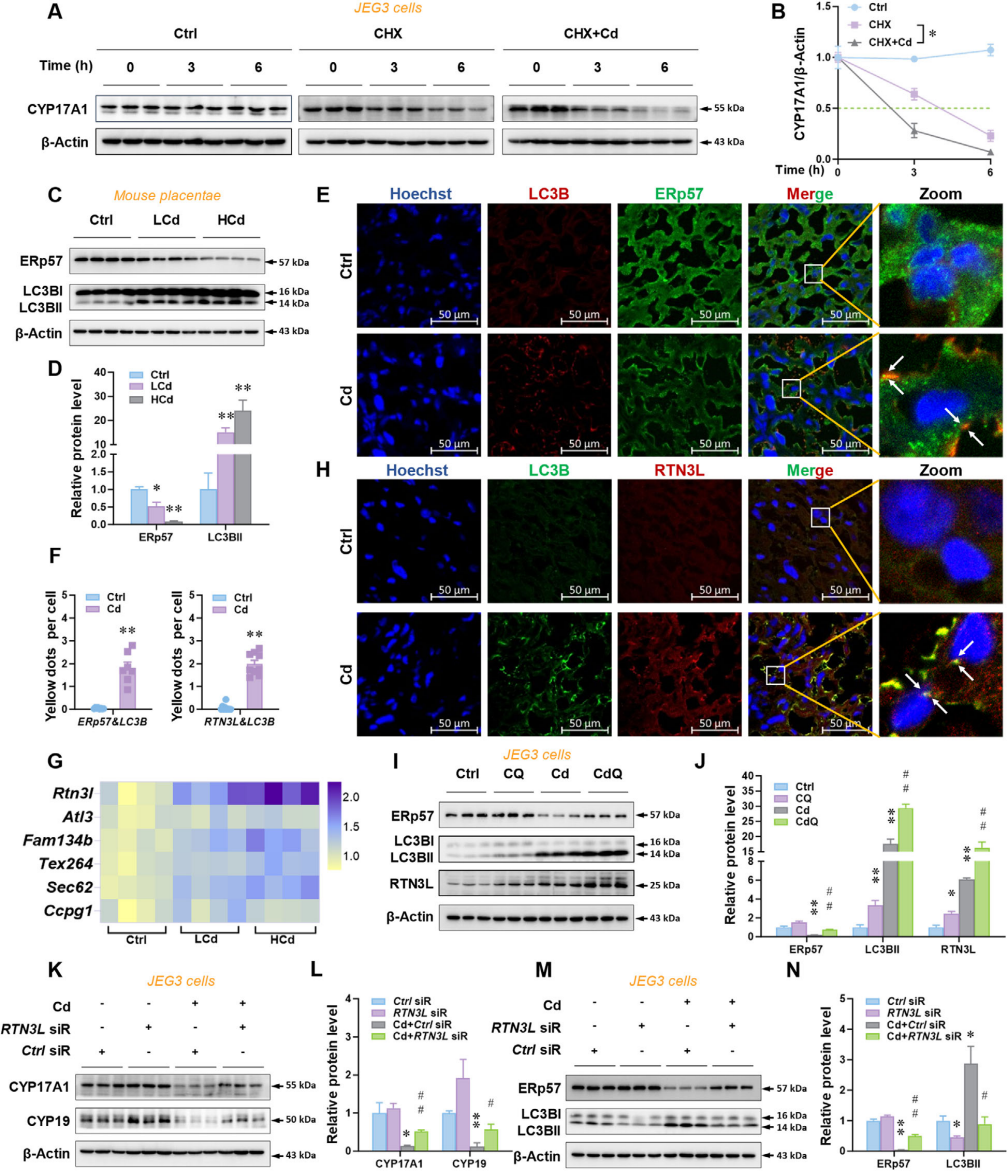
RTN3L‐dependent ER‐phagy activated by environmental stress inhibits estradiol synthesis in mouse placentae. A,B) Degradation of CYP17A1 in JEG3 cells. The JEG3 cells were treated with CHX or CHX and Cd. C–I) The pregnant mice were exposed to low concentrations of CdCl_2_ (50 mg L^−1^, LCd) or high concentrations of CdCl_2_ (150 mg L^−1^, HCd) by drinking water from GD8 to GD16. After euthanasia on GD16, sera, placentae, and fetal testes were collected. C,D) Protein levels of ERp57 and LC3B‐II in mouse placentae. E,F) LC3B and ERp57 immunofluorescent analysis of mouse placentae. Yellow dots: co‐localizations of LC3B with ERp57. Hoechst33258 was used to tag the nucleus. G) mRNA levels of endoplasmic reticulum autophagy receptors in mouse placenta. H,F) LC3B and RTN3L immunofluorescent analysis of mouse placentae. Yellow dots: co‐localizations of LC3B with RTN3L. Hoechst33258 was used to tag the nucleus. I,J) Protein levels of ERp57, LC3B‐II, RTN3L in JEG3 cells. The cells were stimulated with CdCl_2_ after CQ pretreatment. K–N) Protein levels of CYP17A1, CYP19, ERp57, and LC3B‐II in JEG3 cells. The cells were stimulated with CdCl_2_ after *RTN3L* siRNA transfection. All data were analyzed using one‐way *ANOVA* and presented as mean *± SEMs*. *n* = 3–6. ^*^ *p*<0.05, ^**^ *p*<0.01, compared to Ctrl or *Ctrl* siRNA. ^#^ *p*<0.05, ^##^ *p*<0.01, compared to Cd/Cd + *Ctrl* siRNA.

To investigate the mechanism of decreased CYP17A1 and CYP19 caused by environmental stress, human JEG3 cells were exposed to Cd with translation inhibitor Cycloheximide (CHX) treatment. The data displayed that the decay of CYP17A1 protein was increased in CHX + Cd group compared to CHX group (**Figure** **3**A,B). The result suggests that the decrease in placental E2 synthesis may be due to E2 synthetase degradation. CYP17A1 and CYP19 are two E2 synthetases localized in the endoplasmic reticulum (ER). To explore the mechanism of ER CYP17A1 and CYP19 degradation, the level of placental ER‐phagy was detected after Cd exposure. Immunoblotting analysis indicated that ERp57, a marker for ER, was reduced in placentae after Cd exposure (Figure 3C,D. Gestational Cd exposure significantly increased the level of placental LC3B‐II, a marker of autophagosome (Figure 3C,D). Immunofluorescence analysis indicated colocalizations of LC3B and ERp57 in Cd‐exposed placentae were elevated (Figure 3E,F). As above, environmental stress activates ER‐phagy to inhibit E2 synthesis in the placentae.

The mRNA levels of ER‐phagy receptors were up‐regulated in Cd‐exposed placentae (Figure 3G). Although ER‐phagy receptor *Rtn3l*, *Fam134b*, and *Sec62* all exhibited significant increases in mRNA levels under Cd treatment, the fold change in *Rtn3l* mRNA was the highest among the above three mRNAs (*Rtn3l* mRNAs fold change, 2.03; *Fam134b* mRNAs fold change, 1.53; *Sec62* mRNAs fold change, 1.52) (Figure 3G). In addition, there were no dose‐dependent changes in SEC62 and FAM134B expression in Cd‐exposed placentae (Figure S3A—C, Supporting Information). The mRNA and protein levels of RTN3L exhibited dose‐dependent changes in placentae exposed to environmental Cd. (Figure S3D,E, Supporting Information). Immunofluorescence analysis indicated colocalizations of LC3B and RTN3L in Cd‐exposed placentae were much more than in Ctrl group (Figure 3H,F). As expected, the expression of ERp57 in Cd‐treated human JEG3 cells was decreased (Figure S3F,G, Supporting Information). LC3B‐II and RTN3L expression in Cd‐treated cells was also increased (Figure S3F,G, Supporting Information). In contrast to the chloroquine (CQ) group, the expression of ERp57 was lower in CdQ (Cd + CQ) group, and the expression of LC3B‐II and RTN3L was higher in CdQ group (Figure 3I,J). As mentioned above, environmental stress activates RTN3L‐dependent ER‐phagy, and autophagy flux is unobstructed. To probe the impact of ER‐phagy on Cd‐exposed placental E2 synthetase degradation. The results showed that the co‐localization of RTN3L and CYP17A1 was increased in gestational Cd‐exposed placentae (Figure S3H,I, Supporting Information). Moreover, the results revealed that the expression of CYP17A1, CYP19, and ERp57 was recovered in Cd + *RTN3L* siR group. The expression of LC3B‐II and RTN3L was reversed in Cd + *RTN3L* siR group (Figures 3K–N,J,K, Supporting Information). Overall, environmental stress activates RTN3L‐dependent ER‐phagy to inhibit E2 synthesis in mouse placentae.

### 2.4 Human Placental ER‐Phagy Activation is Associated with the Inhibition of Estradiol Synthesis Upon Environmental Stress

**Figure 4:**
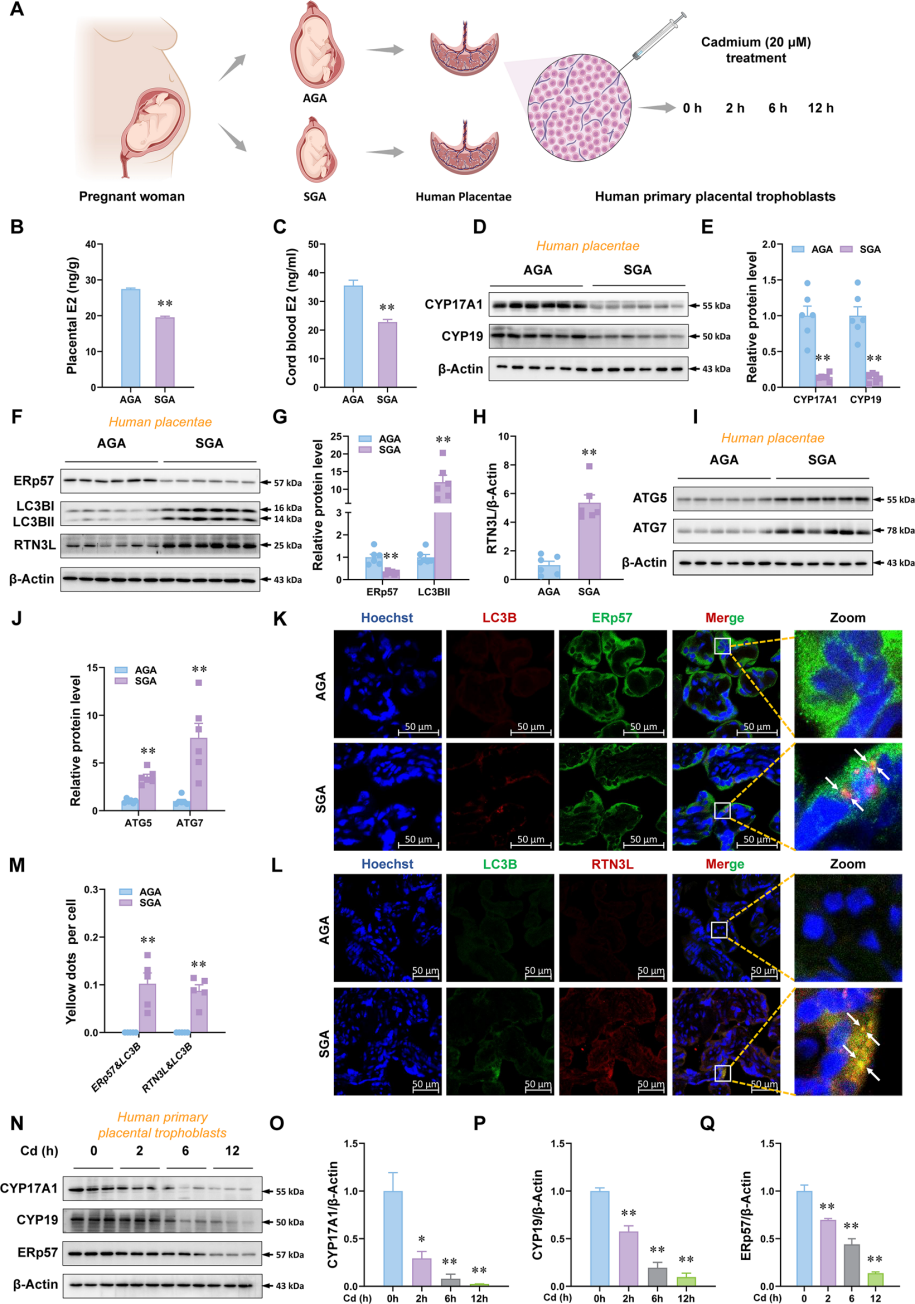
Human placental ER‐phagy activation is associated with the inhibition of estradiol synthesis upon environmental stress. A–M) The human placentae were divided into AGA and SGA. A) Human placenta case‐control study collection diagram. B) Estradiol levels in human placentae. Estradiol levels are nanograms per gram of placentae. C) Estradiol levels in human cord blood. Estradiol levels are nanograms per milliliter of blood. D,E) Protein levels of CYP17A1 and CYP19 in human placentae. F–H) Protein levels of ERp57, LC3B‐II, and RTN3L in human placentae. I,J) Protein levels of ATG5 and ATG7 in human placentae. K–M) LC3B and ERp57 or LC3B and RTN3L immunofluorescent analysis of human placentae. Yellow dots: co‐localizations of LC3B with ERp57 or LC3B with RTN3L. Hoechst33258 was used to tag the nucleus. N–Q) Protein levels of CYP17A1, CYP19, and ERp57 in human primary placental trophoblast cells. The cells were treated with CdCl_2_. All data were analyzed using one‐way *ANOVA* and presented as mean *± SEMs*. *n* = 3–12. ^*^ *p*<0.05, ^**^ *p*<0.01, compared to 0 h or AGA.

E2 levels in human placentae and cord blood were reduced in SGA group (**Figure** **4**B,C). The expression of CYP17A1, CYP19, and ERp57 in SGA group was less than in AGA group (Figure 4D,E). As predicted, the expression of autophagy protein expression, including LC3B‐II, RTN3L, ATG5, and ATG7, was increased in SGA group (Figure 4F–J). The colocalizations of LC3B and ERp57 or RTN3L were also enhanced in SGA group (Figure 4K–M). Moreover, we discovered that the expression of CYP17A1, CYP19, and ERp57 was decreased in human primary placental trophoblasts with Cd treatment (Figure 4N–Q). In summary, human placental ER‐phagy activation is associated with the inhibition of E2 synthesis upon environmental stress.

### 2.5 Placental RTN3L Knockdown Restores Fetal Testicular Development Dysplasia Upon Environmental Stress

**Figure 5:**
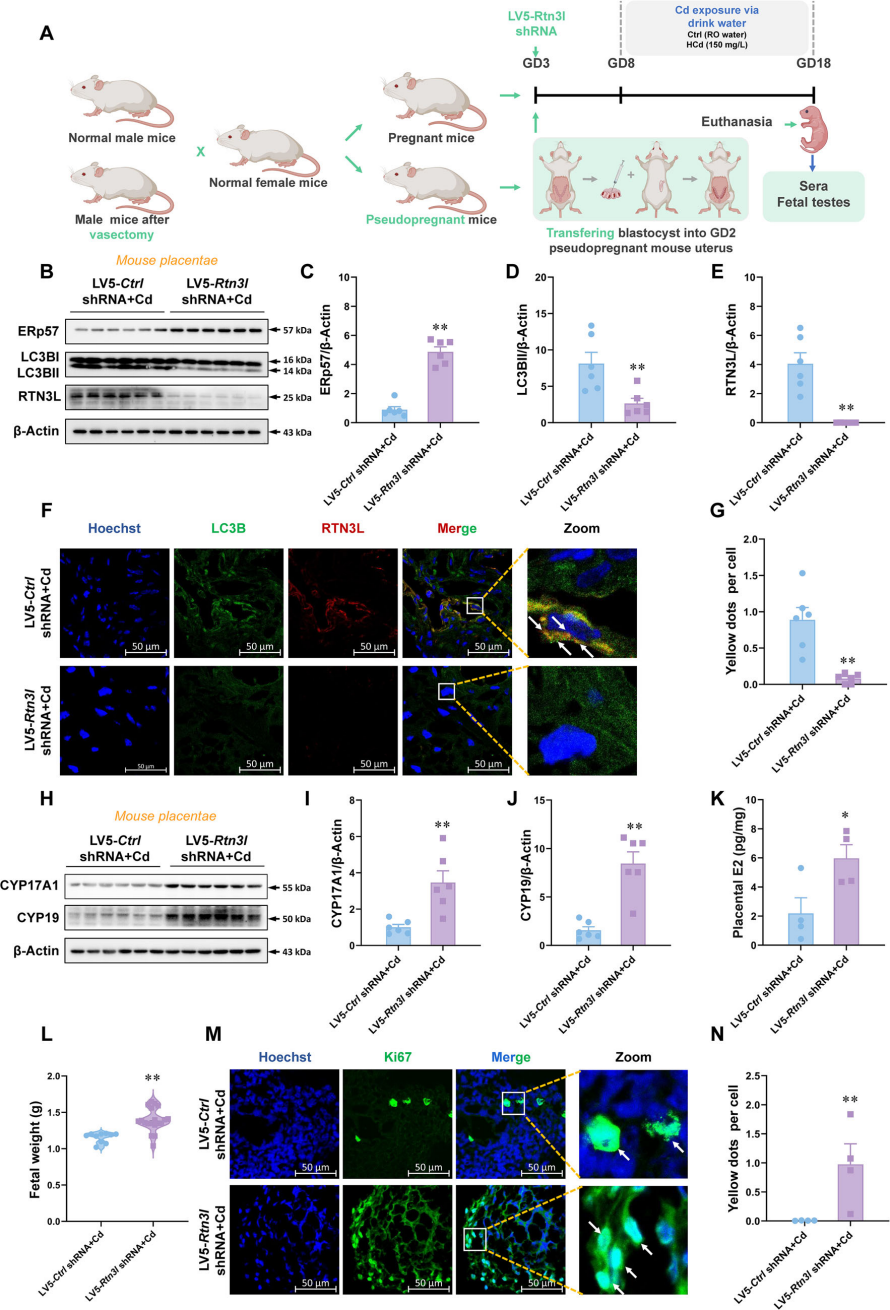
Placental RTN3L knockdown restores fetal testicular development dysplasia upon environmental stress. A–G) After euthanizing GD3 pregnant mice, the blastocysts were removed, infected with LV5 *Rtn3l* shRNA, and suppressed back into the uterus of GD2 pseudopregnant mice. High concentrations of CdCl_2_ (150 mg L^−1^, Cd) for drinking on GD8. Mice were euthanized on GD18, and sera, placentae, and fetal testes were collected. A) Animal experiment diagram of blastocyst lentivirus transfection. B–E) Protein levels of ERp57, LC3B‐II, and RTN3L in mouse placentae. F,G) LC3B and RTN3L immunofluorescent analysis of mouse placentae. Yellow dots: co‐localizations of LC3B with RTN3L. Hoechst33258 was used to tag the nucleus. H–J) Protein levels of CYP17A1 and CYP19 in mouse placentae. K) Estradiol levels in mouse placentae. Estradiol levels are pico per milligram of placentae. L) Male fetal weight. M,N) Ki67 immunostaining of fetal testes. Hoechst33258 was used to tag the nucleus. All data were analyzed using one‐way *ANOVA* and presented as mean *± SEMs*. *n* = 6. ^**^ *p*<0.01, compared to LV5 *Ctrl* shRNA + Cd.

To investigate the role of RTN3L‐dependent ER‐phagy activated by maternal environmental stress during pregnancy in fetal testicular development injury. Placental *Rtn3l* was knocked out by blastocyst transfection with LV5‐*Rtn3l* shRNA (**Figure** **5**A). The results revealed that protein expression levels of ERp57, LC3B‐II and RTN3L were reversed in LV5‐*Rtn3l* shRNA + Cd group compared with LV5‐*Ctrl* shRNA + Cd group (Figure 5B–E). Immunofluorescence co‐localization of LC3B and RTN3L in placentae of LV5‐*Rtn3l* shRNA + Cd group was reduced (Figure 5F,E). It was suggested that *Rtn3l* knockout mice were successfully constructed by blastocyst transfection of LV5‐*Rtn3l* shRNA. Western blotting analysis displayed that the expression levels of placental E2 synthetases, CYP17A1 and CYP19, of pregnant mice transfected with LV5‐*Rtn3l* shRNA and then exposed to Cd were higher than those in the LV5‐*Ctrl* shRNA + Cd group (Figure 5H–J). Meanwhile, ELISA results showed a recovery of E2 levels in placentae in LV5*‐Rtn3l* shRNA + Cd group (Figure 5K). This suggests that maternal environmental stress exposure during pregnancy inhibits E2 synthesis by activating RTN3L‐dependent ER‐phagy. Interestingly, male fetal weight after transfection with LV5‐*Rtn3l* shRNA was higher than that of LV5‐*Ctrl* shRNA + Cd group (Figure 5L). Immunofluorescence analysis showed that the number of Ki67^+^ cells in fetal testis exposed to Cd after blastocyst transfection with LV5‐*Rtn3l* shRNA was increased (Figure 5M,N). In addition, we re‐constructed placental *Rtn3l* knockout mice by injecting LV5‐*Rtn3l* shRNA into GD14 placentae. Western blotting analysis showed that the expression levels of proliferation indicators PCNA and CyclinD1 and E2 receptor ER‐β in the testis of LV5‐*Rtn3l* shRNA + Cd group were increased (Figure S4A—D, Supporting Information). In summary, maternal environmental stress during pregnancy activates RTN3L‐dependent ER‐phagy, leading to fetal testicular development damage.

### 2.6 m6A Modification Promoted by Environmental Stress to Activates Placental RTN3L‐Dependent ER‐Phagy

**Figure 6:**
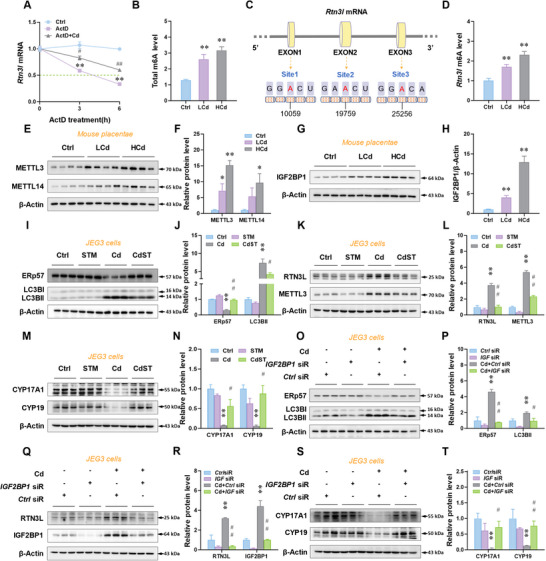
m6A modification promoted by environmental stress to activate placental RTN3L‐dependent ER‐phagy. A) Degradation of *RTN3L* mRNA in JEG3 cells. The cells were stimulated with CdCl_2_ after ActD pretreatment. B–H) The pregnant mice were exposed to low concentrations of CdCl_2_ (50 mg L^−1^, LCd) or high concentrations of CdCl_2_ (150 mg L^−1^, HCd) by drinking water from GD8 to GD16. After euthanasia on GD16, sera, placentae, and fetal testes were collected. B) Total m6A level in mouse placentae mRNA. C) m6A modification site on *Rtn3l* mRNA D) m6A level in mouse placentae *Rtn3l* mRNA. E,F) Protein levels of METTL3 and METTL14 in mouse placentae. G,H) Protein levels of IGF2BP1 in mouse placentae. I–N) The JEG3 cells were stimulated with CdCl_2_ after STM2457 pretreatment. I,J) Protein levels of ERp57 and LC3B‐II in cells. K,L) Protein levels of RTN3L and METTL3 in cells. M,N) Protein levels of CYP17A1 and CYP19 in cells. O–T) The JEG3 cells were stimulated with CdCl_2_ after *IGF2BP1* siRNA transfection. O,P) Protein levels of ERp57 and LC3B‐II in cells. Q,R) Protein levels of RTN3L and IGF2BP1. S,T) Protein levels of CYP17A1 and CYP19 in cells. All data were analyzed using one‐way *ANOVA* and presented as mean *± SEMs*. *n* = 3–4. ^*^ *p*<0.05, ^**^ *p*<0.01, compared to Ctrl or *Ctrl* siRNA. ^#^ *p*<0.05, ^##^ *p*<0.01, compared to Cd or Cd + *Ctrl* siRNA.

To explore the mechanism by which Cd up‐regulates the expression of RTN3L, actinomycin D (ActD), a transcription inhibitor, was used to treat JEG3 cells. Cd exposure significantly repressed the decay of *RTN3L* mRNA in the presence of ActD (**Figure** **6**A). Further data indicated that gestational Cd exposure increased the level of m6A modification in placentae (Figure 6B). SARMP database analysis indicated that there were several high‐confidence m6A binding sites in *Rtn3l* mRNA (Figure 6C). MeRIP‐PCR analysis confirmed that gestational Cd exposure increased the level of m6A‐methylated *Rtn3l* mRNA in placentae (Figure 6D). We used the SRAMP database to predict that there are six potential m6A‐methylated sites with very high confidence (Figure S5A, Supporting Information). The MeRIP‐PCR analysis was then performed and indicated that the levels of m6A modification in sites 1, 4, and 5 of *Rtn3l* mRNA in Cd‐exposed placentae were significantly increased (Figure S5B, Supporting Information). The Spearman correlation analysis speculated that m6A modification site 1 of *Rtn3l* mRNA was the most important (Figure S5C, Supporting Information). To explore its mechanism, we used the RM2Target database, a comprehensive database for targets of writers, erasers, and readers of RNA modifications, and obtained potential writers (METTL3 and METTL14) and readers (IGF2BP1, IGF2BP2, and IGF2BP3) of at gestational Cd exposure increased the level of m6A‐methylated *Rtn3l*. Our results confirmed that the protein expressions of METTL3, METTL14, and IGF2BP1 were increased in Cd‐exposed placentae (Figures 6E–H and S5D–G, Supporting Information). In vitro experiments further verified the above conclusion (Figure S5H,I, Supporting Information). Increased METTL3, METTL14, and IGF2BP1 protein levels were also observed in human SGA placentae (Figure S5J,K, Supporting Information). In brief, environmental stress promotes *Rtn3l* mRNA m6A modification and increases its stability.

To investigate the role of m6A modification on ER‐phagy activation and E2 synthesis inhibition upon gestational stress exposure, we used STM2457, an inhibitor for METTL3, to treat human JEG3 cells. STM2457 obviously blocked Cd‐induced RTN3L‐dependent ER‐phagy activation, as evidenced by the increment in ERp57, and the reduction in RTN3L and LC3B‐II (Figure 6I–L). Expectedly, STM2457 also alleviated Cd‐induced degradation of CYP17A1 and CYP19 proteins (Figure 6 M,N). In addition, the results showed that the protein level of RTN3L in *METTL3*‐knockdown JEG‐3 cells was decreased after Cd exposure (Figure S5L,M, Supporting Information). *IGF2BP1*‐knockdown cells were generated via *IGF2BP1* siRNA transfection. Similarly, *IGF2BP1* knockdown also reversed Cd‐caused ER‐phagy activation and E2 synthesis inhibition in human placental JEG3 cells (Figure 6O–T). In summary, environmental stress promotes m6A modification to activate RTN3L‐dependent ER‐phagy in placentae.

### 2.7 Gestational SAH Supplementation Reverses Fetal Mouse Testicular Dysplasia Upon Environmental Stress

**Figure 7:**
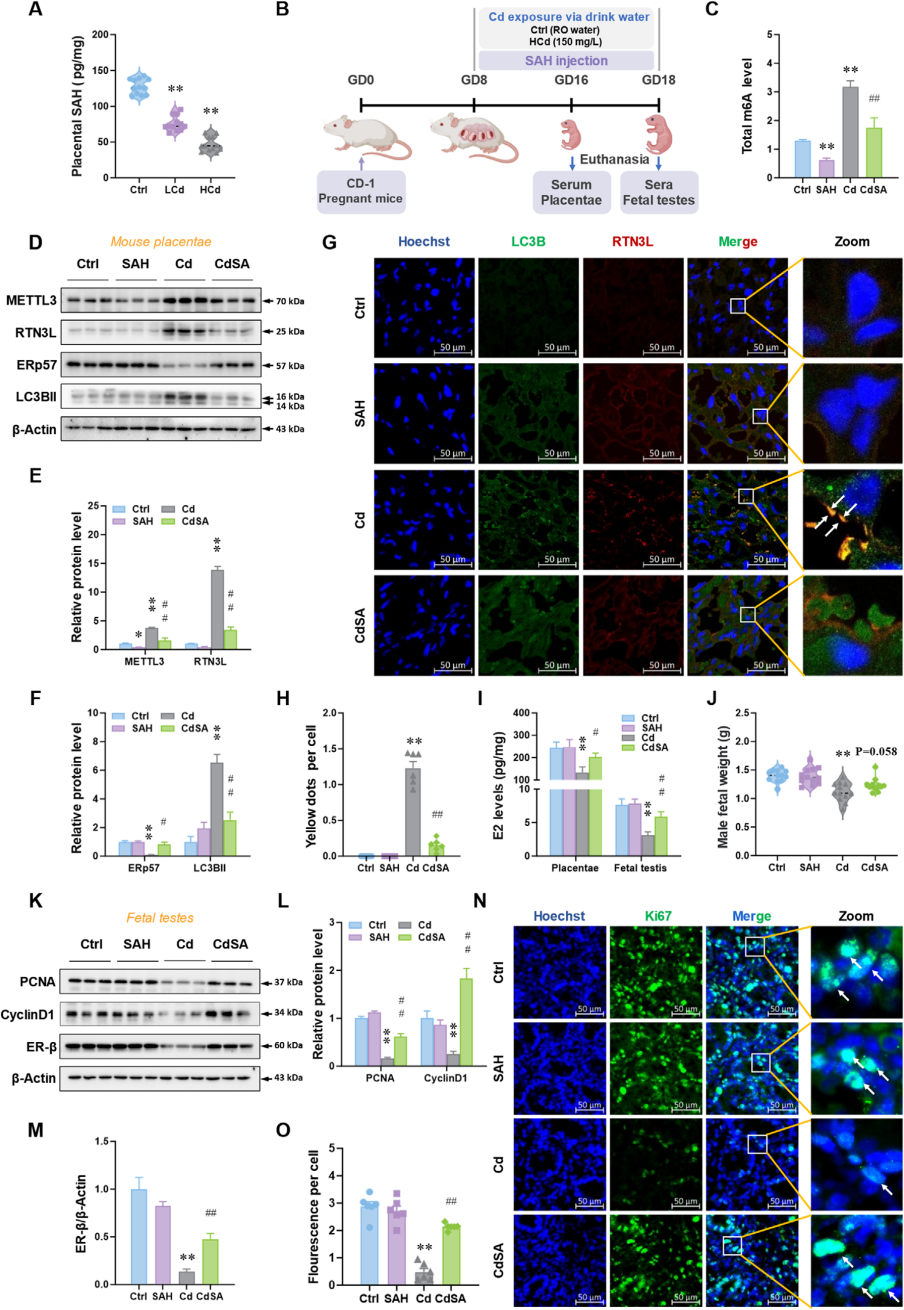
Gestational SAH supplementation reverses fetal mouse testicular dysplasia upon environmental stress. A) S‐adenosylhomocysteine (SAH) levels in placentae. The pregnant mice were exposed to low concentrations of CdCl_2_ (50 mg L^−1^, LCd) or high concentrations of CdCl_2_ (150 mg L^−1^, HCd) by drinking water from GD8 to GD16. After euthanasia on GD16, sera, placentae, and fetal testes were collected. B–O) The pregnant mice were exposed to high concentrations of CdCl_2_ (150 mg L^−1^, Cd) by drinking water paralleled with SAH intervention from GD8 to GD16 or GD18. After euthanasia on GD16 or GD18, sera, placentae, and fetal testes were collected on GD16 or GD18. B) Animal experiment diagram of SAH intervention. C) Total m6A level in mouse placentae mRNA. D–F) Protein levels of METTL3, RTN3L, ERp57, and LC3B‐II in mouse placentae. G,H) LC3B and RTN3L immunofluorescent analysis of mouse placentae. Yellow dots: co‐localizations of LC3B with RTN3L. Hoechst33258 was used to tag the nucleus. I) Estradiol levels in mouse placentae or fetal testes. Estradiol levels are pico per milligram of placentae or testes. J) Male fetal weight. K–M) Protein levels of PCNA, CyclinD1, and ER‐β in fetal testes. N,O) Ki67 immunofluorescent analysis of fetal testes. Hoechst33258 was used to tag the nucleus. All data were analyzed using one‐way *ANOVA* and presented as mean *± SEMs*. *n* = 3–12. ^*^ *p*<0.05, ^**^ *p*<0.01, compared to Ctrl. ^#^ *p*<0.05, ^##^ *p*<0.01, compared to Cd.

S‐adenosylhomocysteine (SAH), an intermediate metabolite in the methionine cycle, is an inhibitor of METTL3‐METTL14 heterodimer complex. To explore the mechanism by which environmental stress enhanced m6A modification, SAH content was detected in placentae. The SAH levels were decreased in placentae after Cd exposure (**Figure** **7**A). Moreover, we gave pregnant mice SAH intervention while Cd exposure (Figure 7B). SAH supplementation decreased total m6A modification levels and METTL3 protein expression in the placenta (Figure 7C–E). ER‐phagy activation caused by Cd exposure was also repressed in the presence of SAH supplementation (Figure 7D–F). Furthermore, immunofluorescence analysis indicated that colocalizations of LC3B and RTN3L in Cd group were more than that in CdSA (SAH + Cd) group (Figure 7G,H). SAH supplements also reversed Cd‐induced reduction of E2 levels in placentae and fetal testes (Figure 7I). The results showed that exogenous SAH supplements restored the decline in male fetal weight induced by prenatal stress (Figure 7J). Compared to Cd group, the levels of PCNA, CyclinD1 and ER‐β in CdSA group were higher in fetal testes (Figure 7K–M). As speculated, exogenous SAH supplement alleviated Cd‐evoked reduction of the number of Ki67^+^ cells in fetal testes (Figure 7N,O). Overall, the gestational SAH supplement reverses Cd‐induced ER‐phagy and E2 synthesis repression via inhibiting m6A modification in placentae.

## 3 Discussion

Male infertility accounts for 30–50% of infertility cases worldwide.^[^ ^44^ ^]^ Fetal testicular dysplasia leads to adult male infertility. Previous studies have shown that developmental environmental stress exposure damages fetal testicular development, subsequently causing adult male infertility.^[^ ^6^, ^7^ ^]^ Cadmium (Cd), a classical environmental stressor, is known to be toxic to reproductive development.^[^ ^12^ ^]^ In this study, an animal model of fetal testicular dysplasia was generated via developmental environmental Cd exposure. We found that gestational Cd exposure impaired fetal testicular development. However, the underlying mechanism remains unclear. Estradiol (E2), a primary form of estrogen, is an indispensable hormone that promotes cell proliferation and fetal testicular development. Previous studies have reported that maternal environmental stress exposure impairs fetal brain and fetal liver development by blocking the E2/ER signaling.^[^ ^25^, ^32^ ^]^ In this study, the E2/ ER‐β signaling was also repressed in dysplastic fetal testes. Importantly, maternal E2 supplementation significantly alleviated environmental stress‐induced fetal testicular dysplasia. Further data also confirmed that maternal E2 supplementation activated E2/ER signaling in dysplastic fetal testes. Therefore, maternal environmental stress during gestation damages fetal testicular development via blocking E2/ER signaling.

Fetal testicular E2 has three sources, maternal ovaries, placentae, and fetal testes.^[^ ^21^, ^22^ ^]^ Ovariectomy performed on pregnant mice clarified the role of ovary‐derived E2 in environmental stress‐induced fetal testicular dysplasia. There were no significant differences in dysplastic fetal testes after ovariectomy. Furthermore, the changes in E2 synthetases were not in line with the changes in fetal testicular dysplasia. As above, maternal ovary‐derived and fetal testis‐derived E2 has no role in environment stress‐induced fetal testicular dysplasia. The placenta is an important organ connecting mothers and fetuses. In the mid‐late stages of pregnancy, the placenta replaces the ovaries as the main organ for E2 secretion.^[^ ^23^, ^24^, ^45^ ^]^ Environmental stressor Cd is extremely difficult to cross the placental barrier and accumulates in placentae, suggesting placenta may be the target for the developmental toxicity of Cd.^[^ ^46^ ^]^ In the current study, the ratio of fetal weight to placental weight was reduced after environmental stress, suggesting that placental function might be damaged. The placenta was the main organ of estradiol synthesis rather than estradiol decomposition during pregnancy.^[^ ^24^ ^]^ Based on the phenotype of the decrease in placental estradiol, this study aimed to investigate the effects of gestational Cd exposure on placental estradiol synthesis. Cholesterol was the raw material for estradiol synthesis.^[^ ^47^ ^]^ Our recent study revealed the level of placental cholesterol was increased in environmental Cd‐exposed pregnant mice,^[^ ^48^ ^]^ indicating that the decrease in placental estradiol was not attributed to the decrease in raw material cholesterol. Therefore, we further explore the effect of environmental Cd exposure on placental estradiol synthetases. RT‐qPCR results showed that Cd exposure during pregnancy did not change mRNA levels of estrogen synthetases *Cyp17a1* and *Cyp19*. However, our previous studies showed that Cd exposure during pregnancy reduced the protein expression of CYP17A1 and CYP19.^[^ ^25^, ^32^ ^]^ These results suggested that Cd exposure might promoted the degradation of placental CYP17A1 and CYP19. Further results of the human study showed that E2 levels in human placentae and cord blood were reduced in SGA group. CYP17A1 and CYP19 protein levels in SGA group were decreased. Moreover, we discovered that in human primary placental trophoblasts, the expression of CYP17A1 and CYP19 was decreased with Cd treatment. As above animal and human study results, gestational environmental stress impairs fetal testicular development by inhibiting placental E2 synthesis.

To explore the mechanism of reduction in E2 synthases, human JEG‐3 cells were treated with cycloheximide (CHX), an inhibitor of protein translation. Cd exposure accelerated E2 synthetase CYP17A1 degradation rate in human JEG‐3 cells. CYP17A1 and CYP19 are located in the endoplasmic reticulum (ER).^[^ ^31^ ^]^ ER‐phagy, the process of lysosomes selectively degrading ER, leads to ER protein loss.^[^ ^26^, ^28^ ^]^ The expression of ER‐phagy proteins was changed in Cd‐exposed placentae. ER‐phagy is mediated by various ER‐phagy receptors, such as RTN3L, SEC62, and FAM134B. Gestational Cd exposure significantly upregulated placental RTN3L expression. Moreover, colocalization of LC3B‐II and RTN3L was also increased. The degradation in ER was blocked in Cd‐treated human JEG‐3 cells after lysosome inhibitor CQ treatment, suggesting that Cd activated the flow of RTN3L‐dependent ER‐phagy. The inhibition of E2 synthesis and the activation of RTN3L‐dependent ER‐phagy also occurred in SGA placentae and Cd‐administrated human placental primary trophoblasts. Based on lentivirus transfection and embryo transplantation, placenta‐specific knockout gene mice could be constructed.^[^ ^49^, ^50^ ^]^ Mice with placenta‐specific *Rtn3l*‐knockout were generated to investigate the role of RTN3L‐dependent ER‐phagy in Cd‐induced placental E2 synthesis inhibition. After placenta‐specific *Rtn3l* knockout, placental E2 synthesis was increased in Cd‐exposed pregnant mice. Placental *Rtn3l* knockout also restored Cd‐caused fetal testicular dysplasia. On the other hand, it has been reported that placental spot injection could also construct mice with placenta‐specific gene knockout.^[^ ^51^ ^]^ Another kind of placenta‐specific *Rtn3l* knockout mouse was constructed based on this method, and it confirmed previous study results. Moreover, the results of the human study showed that human placental ER‐phagy was activated in SGA group. Cd exposure also activated ER‐phagy in human primary placental trophoblast cells. As above, gestational environmental stress inhibits placental E2 synthesis via activating RTN3L‐dependent ER‐phagy.

Protein homeostasis was maintained by its synthesis and degradation.^[^ ^52^ ^]^ Based on the results of immunofluorescence and CQ‐treated cells, gestational Cd exposure activated ER‐phagy to degrade RTN3L protein in placentae. However, the phenotype of RTN3L protein was increased in Cd‐exposed mouse placentae and human placental trophoblasts. As above, the synthesis rate of RTN3L was higher than the rate of degradation. Expectedly, the increase in *Rtn3l* mRNA was observed in Cd‐treated mouse placentae. We used actinomycin D to inhibit gene transcription and showed that Cd exposure enhanced *RTN3L* mRNA stability in human placental trophoblast cells. m6A was known to be the most common internal modification on mRNA.^[^ ^53^ ^]^ Moreover, a previous study indicated that placental m6A modification promoted mRNA stability.^[^ ^54^ ^]^ Therefore, the current study focused on the effect of m6A modification on placental RTN3L expression following gestational Cd exposure.

N6‐methyladenosine (m6A) modification, the most common internal modification in eukaryotic mRNA, plays an important role in regulating autophagy.^[^ ^34^, ^43^ ^]^ Gestational Cd exposure significantly upregulated the expression of m6A writers METTL3 and METTL14, thereby increasing the level of total m6A in placentae. According to SRAMP database, it was found that *Rtn3l* mRNA has multiple high‐confidence m6A modification sites. MeRIP‐qPCR analysis confirmed that maternal Cd exposure upregulated the level of m6A methylated *Rtn3l* mRNA in placentae. We used the SRAMP database to predict that there are six potential m6A‐methylated sites with very high confidence. The MeRIP‐PCR analysis was then performed and indicated that the levels of m6A modification in sites 1, 4, and 5 of *Rtn3l* mRNA in Cd‐exposed placentae were significantly increased. We analyzed the correlation between the changing trend of m6A modification in each site and *Rtn3l* mRNA. The Spearman correlation analysis speculated that m6A modification in site 1 of *Rtn3l* mRNA was the most important. STM2457, an inhibitor of METTL3, was used to treat human placental trophoblast cells. The data indicated that STM2457 blocked the activation of Cd‐induced RTN3L‐dependent ER‐phagy. In addition, the results showed that the protein level of RTN3L in *METTL3*‐knockdown JEG‐3 cells was decreased after Cd exposure. In vivo, we used S‐adenosylhomocysteine (SAH), an inhibitor of METTL3‐METTL14 complex, to administrate pregnant mice. The data also indicated that SAH significantly repressed RTN3L‐dependent ER‐phagy and then mitigated fetal testicular developmental disorder in Cd‐exposed group. We treated JEG‐3 cells using Actinomycin D (ActD) to block translation and found the stability of *RTN3L* mRNA was increased in Cd‐stimulated cells. The fate of m6A‐modified mRNA, including splicing, translation, and decay, depends on the function of the reader that recognizes and incorporates m6A‐modified mRNA.^[^ ^55^ ^]^ According to RM2Target website, we found that IGF2BPs combine with *Rtn3l* mRNA. Through ganged immunoblotting, the expression level of IGF2BP1, a reader promoting the stability of m6A‐modified mRNA, was elevated after maternal Cd exposure in placentae. The *IGF2BP1*‐deficient cell was then generated. The data confirmed that *IGF2BP1* deficiency suppressed Cd‐stimulated RTN3L‐dependent ER‐phagy. Therefore, gestational environmental stress activates placental ER‐phagy through increasing m6A modification in *Rtn3l* mRNA.

**Figure 8:**
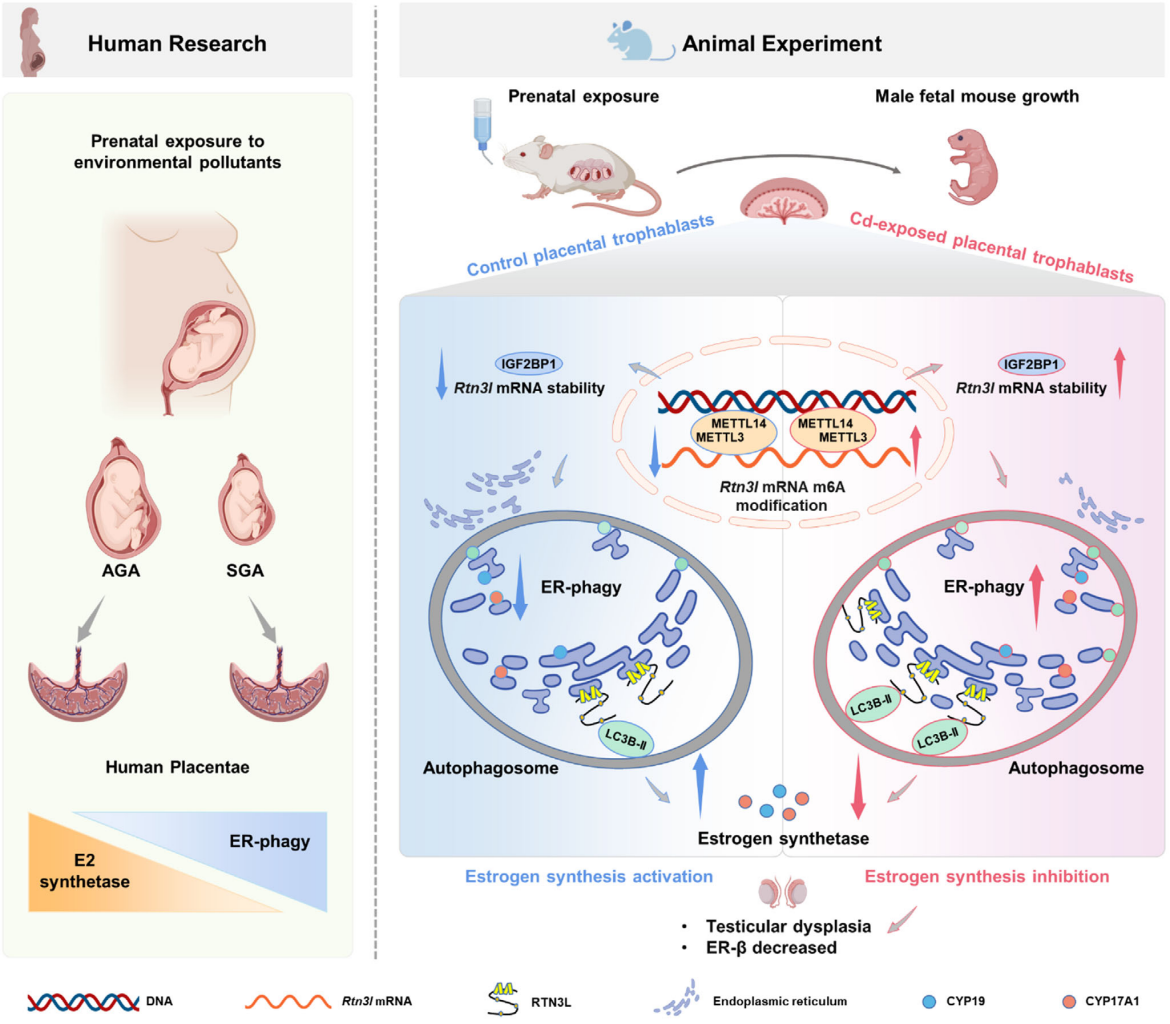
Placental RTN3L‐dependent ER‐phagy contributes to fetal testicular dysplasia upon environmental stress.

In summary, this study reveals that gestational environmental stress inhibits placental E2 synthesis, leading to fetal testicular dysplasia. RTN3L‐dependent ER‐phagy is involved in gestational environmental stress‐inhibited placental E2 synthesis. Further, m6A modification contributes to placental *Rtn3l* mRNA stability increasing upon maternal environmental stress during pregnancy. Therefore, placental RTN3L‐dependent ER‐phagy contributes to fetal testicular dysplasia upon environmental stress (**Figure** **8**). This study demonstrates the early prevention and treatment of adult male infertility from the perspective of fetal‐derived diseases and provides a potential target for future drug discovery.

There are three limitations in the current study. First, we did not explore other mechanisms that up‐regulated the expression of RTN3L in gestational environmental stress‐exposed placentae. Second, only one Endocrine‐Disrupting Chemicals (EDCs) was used as a representative, and additional studies should use other EDCs such as bisphenol A and phthalates to verify whether the findings of this study are a common mechanism by which gestational EDCs expoure induced placental estradiol synthesis inhibition. The last was that we did not compare the contribution of placental oxidative stress, inflammation, and ER‐phagy activation in fetal developmental damage.

## Conflict of Interest

The authors declare no conflict of interest.

## Ethical Approval

We performed the animal experimental procedures in reference to the standard operating procedure (Ethical approval number: LLSC20241571). We performed the collection of biological samples of human origin in reference to the standard operating procedure (Ethical approval number: 83244562).

## Supporting information



Supporting Information

## Data Availability

The data that support the findings of this study are available from the corresponding author upon reasonable request.
