# Supplementary material for: Placental RTN3L‐dependent ER‐Phagy Contributes to Fetal Testicular Dysplasia Upon Environmental Stress
Source: Adv Sci (Weinh). 2025 Apr 26;12(25):2500924. doi: 10.1002/advs.202500924 (PMC12224940; doi:10.1002/advs.202500924)
Supplement: Supplementary file 1 — Supporting Information [file ADVS-12-2500924-s001.pdf]

ADVANCED SCIENCE
Open Access

Supporting Information

for *Adv. Sci.*, DOI 10.1002/advs.202500924

Placental RTN3L-dependent ER-Phagy Contributes to Fetal Testicular Dysplasia Upon Environmental Stress

*Ye-Xin Luo, Hua-Long Zhu, Bin-Bin Huang, Cheng-Fang Sun, Xin-Xin Zhang, Xin-Run Wang, Yi-Fan Hu, Xu-Dong Zhang, Shen-Dong Xu, Huan Zhou, Rui Pan, Wei Chang, Zhi Yuan, Yong-Wei Xiong, Xiao-Feng Xu, Ling-Li Zhao, De-Xiang Xu and Hua Wang**

Supplementary Materials

Placental RTN3L-dependent ER-phagy contributes to fetal testicular dysplasia upon environmental stress

Ye-Xin Luo† [1, 2], Hua-Long Zhu† [1,2,3], Bin-Bin Huang† [5], Cheng-Fang Sun [1,2,4], Xin-Xin Zhang [1,2], Xin-Run Wang [1,2], Yi-Fan Hu [1,2,4], Xu-Dong Zhang [1,2], Shen-Dong Xu [1,2], Huan Zhou [1,2], Rui Pan [1,2], Wei Chang [1,2], Zhi Yuan [1,2], Yong-Wei Xiong [1,2,3], Xiao-Feng Xu [6], Ling-Li Zhao [1,2,3], De-Xiang Xu [1,2,3], Hua Wang * [1,2,3]

Corresponding author: Hua Wang* (wanghuadev@ahmu.edu.cn)

The PDF file includes:

Figures. S1-S5

Figure legends for Figs. S1-S5

Supplementary Materials and Methods

Tables S1 to S5

Figure S1

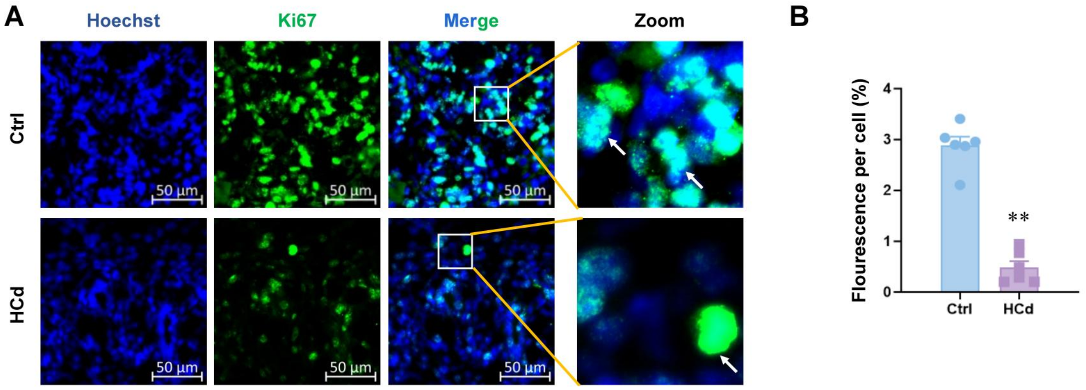

Fig. S1. Prenatal environmental stress inhibits testicular development in fetal mice. (A-B) The pregnant mice were exposed to low doses of $CdCl_2$ (50 mg/L, LCd) or high doses of $CdCl_2$ (150 mg/L, HCd) by drinking water from GD8 to GD18. After euthanasia on GD18, sera, placentae, and fetal testes were collected. (A-B) Ki67 immunofluorescent analysis of fetal testes. Hoechst33258 was used to tag the nucleus. All data were analyzed using one-way *ANOVA* and presented as means ± *SEMs*. $n = 6$. $**P < 0.01$, compared to Ctrl.

Figure S2

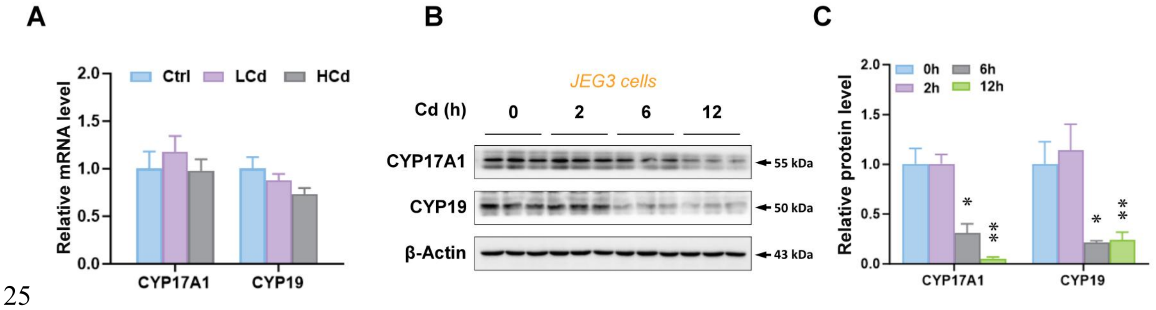

Fig. S2. Prenatal stress inhibits estrogen synthetase synthesis in human placental trophoblastic cells. (A) The pregnant mice were exposed to low concentrations of $CdCl_2$ (50 mg/L, LCd) or high concentrations of $CdCl_2$ (150 mg/L, HCd) by drinking water from GD8 to GD16. After euthanasia, sera, placentae, and fetal testes were collected on GD16. (B-C) Protein levels of CYP17A1 and CYP19 in JEG3 cells. JEG3 cells were treated with $CdCl_2$ for 0 h, 2 h, 6 h, and 12 h, and the medium and cells were collected respectively. All data were analyzed using one-way *ANOVA* and presented as means ± *SEMs*. $n = 3$. $*P < 0.05$, $**P < 0.01$, compared to 0 h.

Figure S3

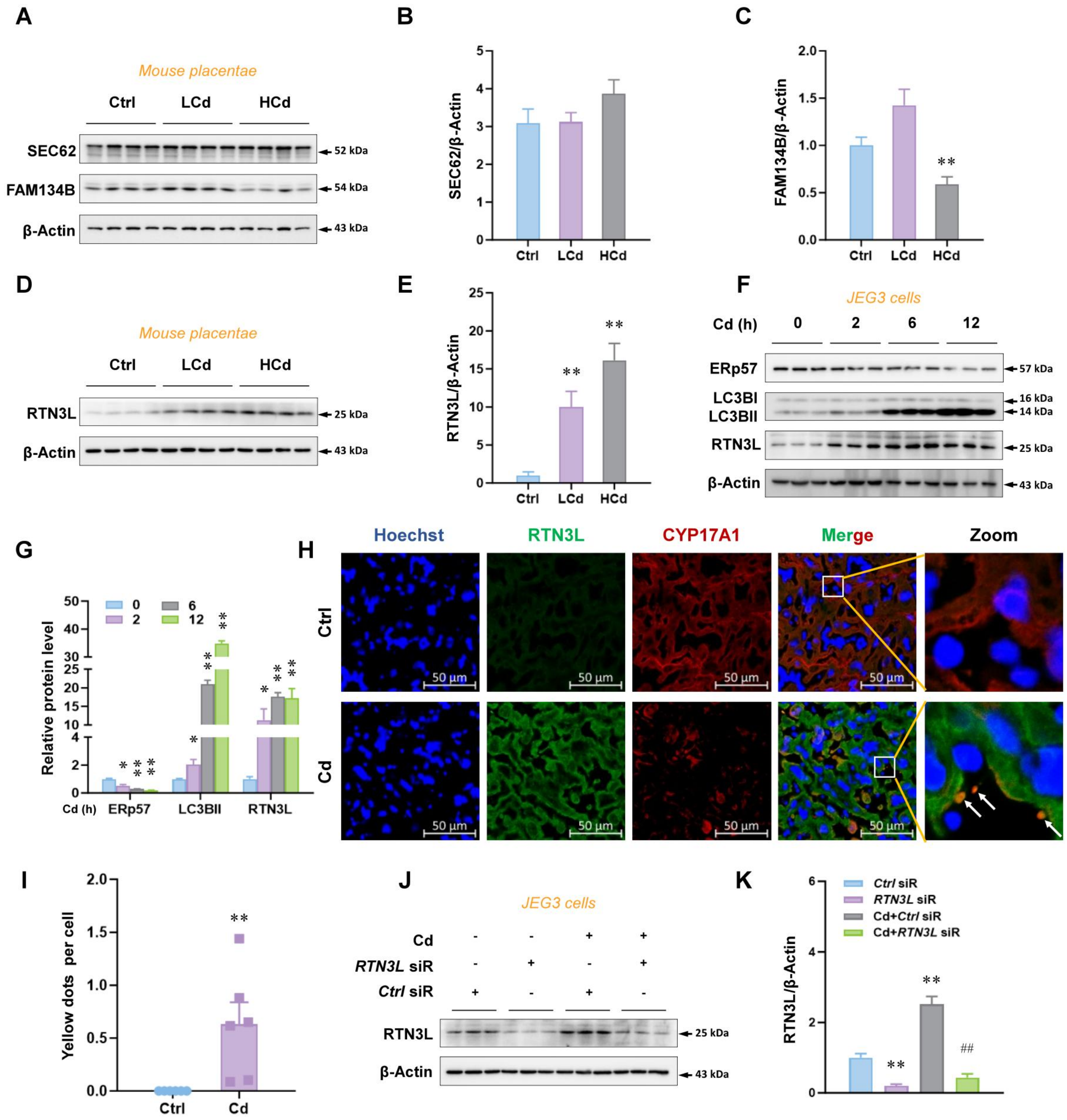

Fig. S3. Prenatal stress activates RTN3L-mediated ER-phagy. (A-E and H-I) The pregnant mice were exposed to low doses of $CdCl_2$ (50 mg/L, LCd) or high doses of $CdCl_2$ (150 mg/L, HCd) by drinking water from GD8 to GD16. After euthanasia on GD16, sera, placentae, and fetal testes were collected. (A-C) Protein levels of SEC62, FAM134B, and RTN3L in mouse placentae. (F-G) JEG3 cells were treated with $CdCl_2$ for 0 h, 2 h, 6 h, and 12 h, and the medium and cells were collected respectively. (F-G) Protein levels of ERp57, LC3BII, and RTN3L in JEG3 cells. (H-I) RTN3L and CYP17A1 immunofluorescent analysis of mouse placentae. Yellow dots: co-localizations of RTN3L with CYP17A1. Hoechst33258 was used to tag the nucleus.

(J-K) Protein levels of RTN3L in JEG3 cells. The cells were stimulated with $CdCl_2$ after *RTN3L* siRNA transfection. All data were analyzed using one-way *ANOVA* and presented as means ± *SEMs*. n = 3-4 *$P < 0.05$, **$P < 0.01$, compared to Ctrl/0 h/*Ctrl* siRNA. $^{##}P < 0.01$, compared to Cd + *Ctrl* siRNA.

Figure S4

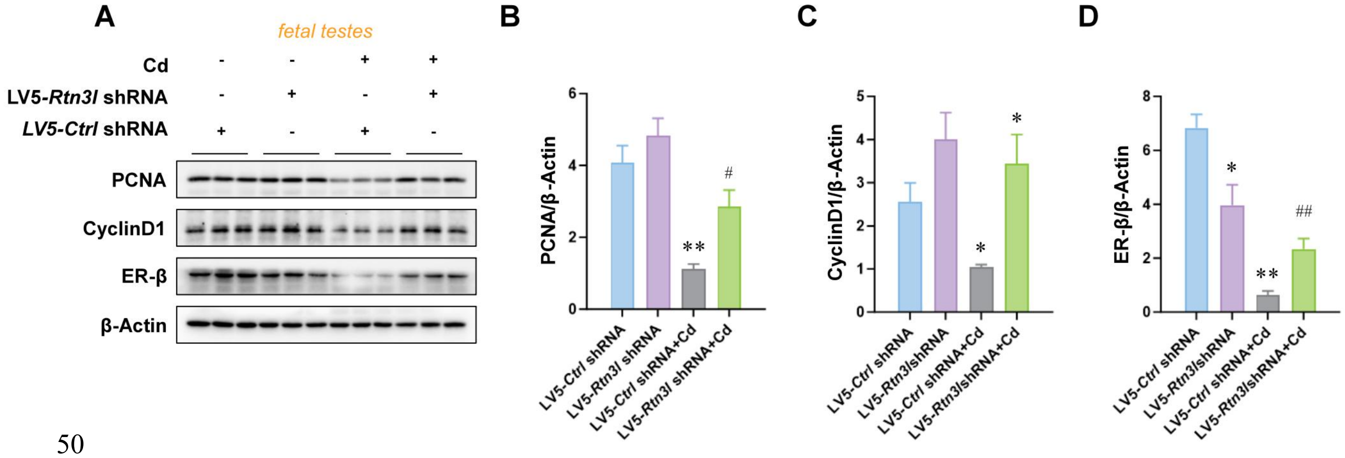

Fig. S4. Placental RTN3L knockdown restores fetal mouse testicular dysplasia upon environmental stress. (A-D) Pregnant mouse GD14 was anesthetized and injected lentivirus into each placenta with sterile 34G Hamilton needles, and the abdomen was sutured and placed in an incubator until the mice recovered. (A-D) Protein levels of PCNA, CyclinD1 and ER-β in fetal testis. All data were analyzed using one-way *ANOVA* and presented as means ± *SEMs*. $n = 3$. $^{*}P < 0.05$, $^{**}P < 0.01$, compared to LV5-*Ctrl* shRNA. $^{\#}P < 0.05$, $^{\#\#}P < 0.01$, compared to LV5-*Ctrl* shRNA + Cd.

Figure S5

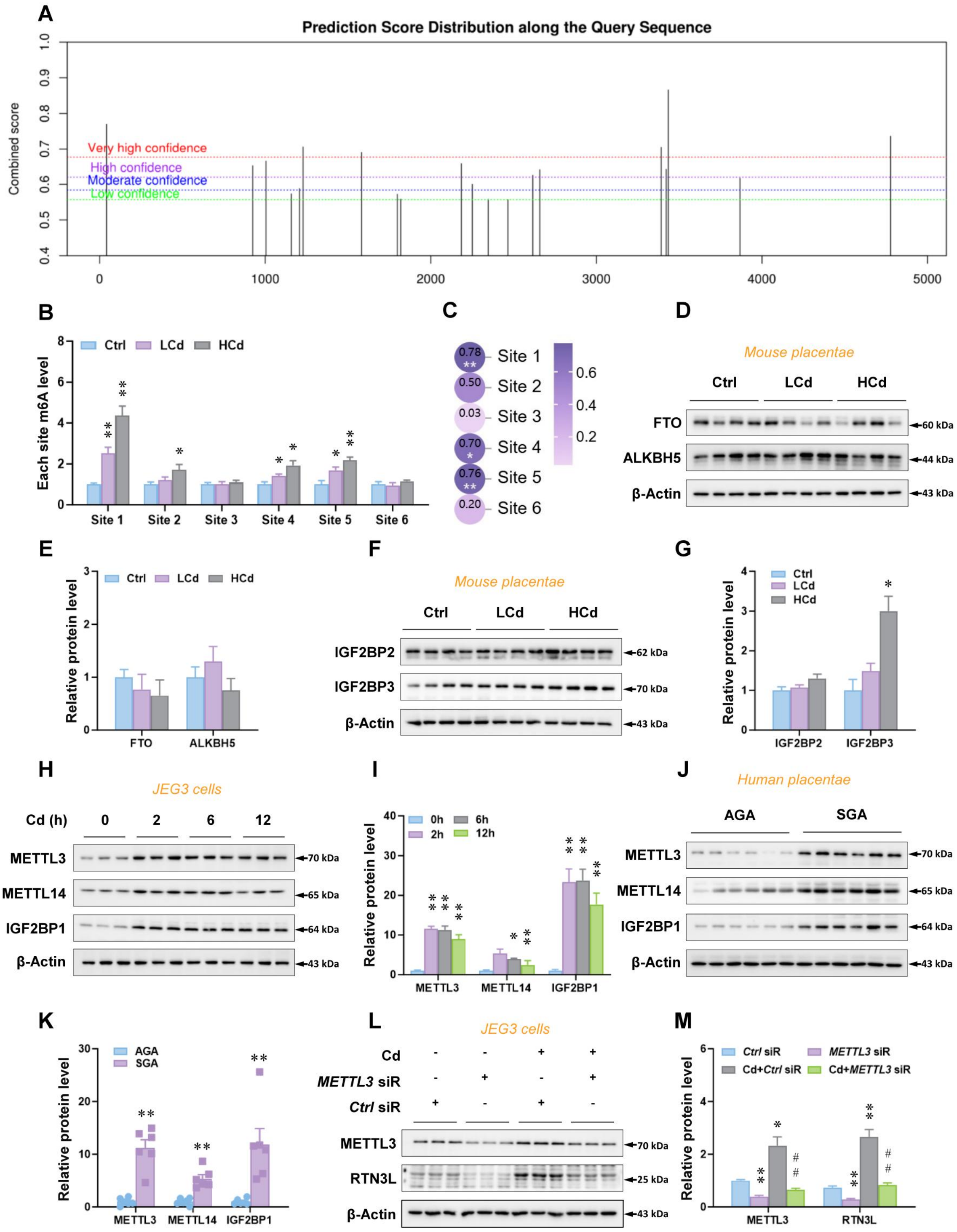

Fig. S5. Prenatal stress enhances m6A modification levels in placentae. (A-G) The pregnant mice were exposed to low doses of $CdCl_2$ (50 mg/L, LCd) or high doses of $CdCl_2$ (150 mg/L, HCd) by drinking water from GD8 to GD16. After euthanasia on GD16, sera, placentae, and fetal testes were collected. (A) Six potential m6A-methylated sites with very high confidence. (B) The m6A levels of predicted sites

in *Rtn3l* mRNA. (C) Visual representation of Spearman correlations among various indices and correlation strength indicated by the color depth. $^{*}P < 0.05$, $^{**}P < 0.01$, $^{***}P < 0.001$. (D-E) Protein levels of FTO and ALKBH5 in mouse placentae. (F-G) Protein levels of IGF2BP2 and IGF2BP3 in mouse placentae. (H-I) Protein levels of METTL3, METTL14, and IGF2BP1 in JEG3 cells. The cells were treated with $CdCl_2$ for 0 h, 2 h, 6 h, and 12 h, and the medium and cells were collected respectively. (J-K) Protein levels of METTL3, METTL14, and IGF2BP1 in human placentae. The human placentae were divided into AGA and SGA. (L-M) Protein levels of METTL3 and RTN3L in JEG3 cells. The cells were stimulated with $CdCl_2$ after *METTL3* siRNA transfection. All data were analyzed using one-way *ANOVA* and presented as means ± *SEMs*. n = 3-6. $^{*}P < 0.05$, $^{**}P < 0.01$, compared to Ctrl/0 h/*Ctrl* siRNA/AGA. $^{\#\#}P < 0.01$, compared to Cd + *Ctrl* siRNA.

Materials and methods

1.1. Reagents

Table 1 Source and Cat No. of the primary antibody.

Antibodies	Source	Cat No.
CYP17A1	Abcam	ab134910
CYP19	Santa Cruz	sc14245
ERp57	Proteintech	15967-1-AP
ATG5	Abcam	ab108327
ATG7	Abcam	ab53255
LC3B	CST	83506
LC3B	CST	43566
PCNA	Santa Cruz	sc7907
CyclinD1	CST	2978s
ERα	Santa Cruz	sc542
ERβ	Santa Cruz	sc8974
RTN3L	Millipore	ABN1723
SEC62	Abcam	ab140644
FAM134B	CST	83414
METTL3	Abcam	ab195352
METTL14	CST	51104S
FTO	Abcam	ab92821
ALKBH5	Abcam	ab195377
IGF2BP1	proteintech	22803-1-AP
IGF2BP2	proteintech	11601-1-AP
IGF2BP3	proteintech	14642-1-AP
Ki67	Abcam	ab1667
β-Actin	CST	4970S

Table 2 Reagent or resource.

Reagents	Source	Cat No.
Cadmium chloride	Sigma-Aldrich	202908
Antibody against N6 -methyladenosine	Synaptic Systems GmbH (Germany)	202003
Actinomycin D	MCE	HY-17559
Cycloheximide	MCE	HY-12320
S-Adenosylhomocysteine	MCE	HY-19528
STM2457	MCE	HY-134836
Chloroquine	MCE	HY-17589A
Meloxicam	MCE	HY-B0261
Cefazolin	MCE	HY-B1892
EmbryoMax Acidic Tyrode’s Solution	Sigma-Aldrich	MR-004-D
EmbryoMax M2 Medium	Sigma-Aldrich	MR-015-D
EmbryoMax Advanced KSOM Embryo Medium	Sigma-Aldrich	MR-101-D

1.2. Animal treatments

8 week-old CD-1 female mice were purchased from Beijing Vital River (Beijing, China). All experimental mice feeding and testing procedures are in accordance with the standard operation formulated by the Experimental Animal Ethics Committee of Anhui Medical University. (1) To investigate whether cadmium exposure during pregnancy induced fetal mice testicular dysplasia, placental estradiol (E2) synthesis inhibition, and RTN3L-dependent ER-phagy activation. The 8-week-old CD-1 female mice mated with 8-week-old CD-1 male mice after one week of adaptive feeding. Gestational day (GD) 0 pregnant rats were randomly divided into three groups according to body weight: control (Ctrl), low cadmium (LCd, 50 mg/L), and high cadmium (HCd, 150 mg/L). LCd and HCd groups drank cadmium water until GD16 or GD18, and the control group was given RO water. (2) In order to investigate whether maternal cadmium exposure during pregnancy can induce abnormal testicular development in fetal mice by decreasing E2 levels. GD0 pregnant mice were randomly divided into four groups according to the body weight distribution method: Ctrl, E2(10 μg/kg), Cd (150 mg/L), and Cd + E2 (CdE). The Cd group and CdE group were exposed to cadmium water to GD18, while the Ctrl group was given RO water. Pregnant mice in the E2 group and CdE group were given intraperitoneal injections of E2 to GD18 on GD8. (3) To investigate whether maternal cadmium exposure during pregnancy inhibits testicular development in fetal mice is not due to decreased E2 in the ovaries, Pregnant rats were divided into a sham-operated control group (Sham), sham-operated + cadmium group (CdS, 150 mg/L), ovariectomy group (OVX) and ovariectomy + cadmium group (CdO, 150 mg/L), Sham and CdS group pregnant mice underwent sham surgery in GD7. The OVX and CdO groups underwent ovariectomy at GD7. The CdS and CdO groups were given cadmium water from GD8 to GD18. (4) To explore the role of RTN3L-dependent ER-phagy in cadmium inhibition of E2 synthesis in pregnant mice. One month after vasectomy of male rats, the ligated male mice and normal male mice were mated with female mice respectively, and pregnant mice and pseudopregnant mice were obtained. GD3 pregnant mice were randomly

divided into two groups according to body weight distribution: LV5- *Ctrl* shRNA + Cd (150 mg/L), and LV5-*Rtn3l* shRNA + Cd (150 mg/L). After euthanizing, the blastocysts were removed, infected with LV5-*Rtn3l* shRNA/LV5-*Ctrl* shRNA and suppressed back into the uterus of GD2 pseudopregnant mice. The LV5- *Ctrl* shRNA + Cd and LV5-*Rtn3l* shRNA + Cd group were exposed to cadmium water from GD8 to GD18. (5) To explore the role of m6A modification in activating ER-phagy in pregnant rats exposed to cadmium. GD0 pregnant rats were randomly divided into four groups according to body weight distribution: Ctrl, S-Adenosylhomocysteine (SAH, 10 mg/kg), Cd (150 mg/L), Cd + SAH (CdSA). Cd and SAD groups were exposed to cadmium water to GD16 or GD18, while the Ctrl group was given RO drinking water. Pregnant mice in SAH group and CdSA group were given intraperitoneal injections of SAH from GD8 to GD18. All the pregnant mouse blood was taken from the heart after anesthesia. Placenta, amniotic fluid, fetal blood, testicles, and other organs were collected.

1.3. Cell culture

JEG-3 cell is from the Chinese Academy of Sciences (Shanghai, China). JEG-3 cells were cultured in the MEM complete medium containing 10% fetal bovine serum, 1% penicillin-streptomycin solution, 1% non-essential amino acids, and 1% sodium pyruvate. JEG-3 cells were grown in an incubator at 37 °C and 5% carbon dioxide. Cell experiments consist of seven parts: (1) To investigate the time-effect relationship between cadmium exposure and E2 synthesis, JEG-3 cells were treated with $CdCl_2$ (20 μM) for 0, 2, 6, and 12 h. (2) To investigate whether cadmium exposure activated ER-phagy, cells were treated with Chloroquine (CQ) for 1 h and then treated with $CdCl_2$ (20 μM) for 12 h. (3) To investigate whether cadmium exposure reduced E2 synthase content, JEG-3 cells were treated with Cycloheximide (CHX) and $CdCl_2$ (20 μM). (4) In order to explore the role of RTN3L-dependent ER-phagy in cadmium-inhibiting E2 synthesis, JEG-3 cells pretreated with *RTN3L* siRNA were treated with $CdCl_2$ (20 μM) for 12 h. (5) To investigate whether cadmium exposure

affected the stability of *RTN3L* mRNA, JEG-3 cells were treated with Actinomycin D (ActD) and $CdCl_2$ (20 μM). (6) In order to investigate the role of m6A modification in cadmium-induced ER-phagy activation of cells, cells were pretreated with STM2457 for 1 h and then treated with $CdCl_2$ (20 μM) for 12 h. (7) To investigate the role of m6A modification in cadmium-induced ER-phagy activation of cells, JEG-3 cells pretreated with *METTL3* siRNA were treated with $CdCl_2$ (20 μM) for 12 h. (8) To investigate the role of IGF2BP1 in cadmium-induced ER-phagy activation of cells, JEG-3 cells pretreated with *IGF2BP1* siRNA were treated with $CdCl_2$ (20 μM) for 12 h.

1.4. Western blotting

The required tissue is fully ground with lysate and quantified to the required concentration. Then add the same amount of sample and marker into the SDS-PAGE hole made in advance. Constant voltage 55 V electrophoresis until marker separation, then constant voltage 110 V electrophoresis to the end. Then, the protein was transferred to the PVDF membrane with a constant current of 200 mA for 2-3.5 h. Next, 5% skim milk prepared with TBST was used to seal the membrane for 1.5 h, the primary antibody was incubated for 1.5 h, and the secondary antibody was incubated for 1.5 h. Finally, the ECL chemiluminescence solution was incubated and the image was displayed on the chemiluminescence imager. All results used β-actin as the internal reference.

1.5. Immunofluorescence

Fresh mouse placentae were immobilized in paraformaldehyde solution for 24 h and soaked with 30% sucrose until the tissues were dehydrated. The dehydrated placenta was cut in half and placed in the center of the inclusion box. After being embedded with OCT, frozen sections were prepared in the frozen microtome. The frozen sections were sealed with a solution of 0.4%Triton100 and 10% donkey serum for 1.5-2 h. The primary antibody mixed with LC3B (1:200) and ERp57 (1:200) or

LC3B (1:200) and RTN3L (1:200) was then incubated at 4℃ overnight. After cleaning with PBS, the corresponding secondary antibody (1:200) was incubated for 2 h. Finally, the nucleus was stained with Hoechst (Sigma, B2883) for 5 minutes. Fluorescence images are taken using confocal microscopy.

1.6. Isolation of total RNA and real-time RT-PCR

The placental or JEG-3 cells were lysed on ice with TRIzol. Then the RNA was extracted by chloroform method, and the RNA was reverse-transcribed into cDNA by Roche reverse transcription kit. The mRNA level of the target gene was detected by Lightcycler®480 real-time fluorescent quantitative PCR (Roche) and 18S was used as a control. The sequence of primers used is shown in Table 3.

Table 3 Primers for Estrogen synthetase real-time RT-PCR or PCR.

Genes	Sequences	Species
Cyp17a1	F: GCCCAAGTCAAAGACACCTAAT	M
	R: GTACCCAGGCGAAGAGAATAGA	
Cyp19	F: ATGTTCTTGGAAATGCTGAACCC	M
	R: AGGACCTGGTATTGAAGACGAG	

Table 4 Primers for real-time RT-PCR or PCR.

Genes	Sequences	Species
Rtn3l	F: AGGTGCCCCTACGATGTCTC	M
	R: GGTTTGCTTGAGTTTTCCTCCA	
Atl3	F: CTGGACTTTATGCTGCGATACTT	M
	R: AGCCTCCTCGCCATGAAAATC	
Fam134b	F: AAACAGCAGAGTCCTGGCAAG	M
	R: AGGTAGCTGAGTATGACCCCA	
Tex264	F: CCAATCCGCAACATAACTGTGG	M
	R: GGGTTGTCATAGTAGACAGCGAT	
Sec62	F: TGATTGCAGTAATAGCAGCCAC	M
	R: GCCCACACTGAGGTAATAAACAC	
Ccpg1	F: AAGCGGCAACTGAAAAGACAA	M
	R: TCCAGCACATTCAAACATACCTG	
RTN3L	F: ACTGGGTTTGTCTTTGGCAC	H
	R: ATGACGGACTTGTAGATCCTGA	

Table 5 Primers for m6A modification sites.

Genes	Sequences	Species
Site 1	F: AACAAGGTTCCAGATGGGGAG	M
	R: ACTGGAAGTTCAGATGGGGAC	
Site 2	F: CAGATGGGGAGGACAGAAGTG	M
	R: ACAAGTCTGCGGGGGATTC	
Site 3	F: TTACCAAATCCACAGGTGACTG	M
	R: CCCCCATCACAGAGTTAGGC	
Site 4	F: CCCAGAGGACTTGATGGCTG	M
	R: TGCAAGAGTTCCACAGGCAA	
Site 5	F: ACAGATGGTTCCTCCCCAGA	M
	R: ATGCAAGAGTTCCACAGGCA	
Site 6	F: CCTGACTCCCTTCCAAGTGC	M
	R: CTTGTGAAGCCACCTCACCT	

1.7. RNA interference

JEG-3 cells were inoculated in a 60mm petri dish until the cell density reached 80%. Lipofectamine 3000 and *RTN3L* siRNA or Lipofectamine 3000 and *IGF2BP1* siRNA were mixed into serum-free Opti-MEM for 15 minutes and then transfected

into petri dishes for 6 h. The culture medium was changed to the complete medium for 36 h, and then $CdCl_2$ (20μM) was added and treated for 12 h to collect cells.

1.8. Assessment of m6A levels

In each hole of the test plate, 80μl binding solution and 2μl diluted positive control were added, and then 200 ng RNA was added to each hole. Mix gently and incubate at 37 ° C for 90 minutes. Wash three times with 150μl cleaning solution. Add 50μl diluted antibody capture solution to each well and incubate at room temperature for 60 min. Then wash each well three times with 150μl cleaning solution. Each well was incubated with 50μl diluted antibody solution at room temperature for 30 minutes, then washed with 150μl cleaning solution four times and then added with 50μl diluted antibody enhanced solution for 30 minutes at room temperature. Last rinse 5 times. Add 100μl developer to each well for 10 minutes to detect sample absorbance at 450nm. So we can calculate the level of m6A in the RNA.

1.9. MeRIP-PCR

mRNA was purified from total placental RNA using Arraystar Seq-Star TM poly(A) mRNA Isolation Kit (Maryland, USA). The purified mRNA was left with 10% as control, and the reaction System was mixed with 2μg Affinity for anti-m6A polyclonal antibody (Synaptic System, 202003) and incubated at 4℃ for 2 h. Then, Dynabeads^{TM} M-280 Sheep anti-Rabbit IgG (TermoFisher, 11204D) magnetic beads were reacted at 4℃ for 2 h. After cleaning with buffer and cleaning buffer, the eluting buffer was added and reacted at 50℃ for 30 minutes, and then chloroform was used to extract the final m6A-modified mRNA.

1.10. RNA stability assay

JEG-3 cells were inoculated into a culture dish and divided into the control group, actinomycin D (5g/mL) group, and Cd plus actinomycin D (5g/mL) group. The cells were lysed by TRIzol at 0 h, 3 h, and 6 h, respectively, and the RNA levels of each

group were detected by qRT-PCR.

1.11. ELISA

The estradiol content in maternal blood, placenta, and fetal testis was determined Estradiol enzyme-linked immunosorbent assay kit was used. After centrifuging the whole blood, serum was retained. Take 50 mg of placenta or fetal testis and grind homogenate in pre-cooled PBS. After centrifugation, leave the supernatant. The estradiol concentration in maternal blood, placenta, and fetal testis was detected by enzymoleter after operation according to the kit instructions.

1.12. Nonsurgical embryo transfer and lentivirus transfection

(1) The male mice were anesthetized and subcutaneously injected with 2mg/kg meloxicam, which was opened in the abdominal midline, and the vas deferens on both sides were ligated and then sutured. Sterile male mice were obtained after one month. (2) Pregnant mice and pseudopregnant mice were obtained by combining normal male mice and sterile male mice with female mice, respectively. (3) The pregnant mice were euthanized in GD3, the uterus was removed, the blastocyst was washed out of the uterine horn with M2 medium, the blastocyst was collected under the stereo microscope, the blastocyst was cleaned with KSOM medium, and the blastocyst zona pellucida was dissolved with hyaluronic acid. Then, the blastocysts were cleaned with M2 and KSOM media and placed in a 5% CO_2 incubator at 37℃. The LV5-*Rtn3l* shRNA and LV5-*Ctrl* shRNA were diluted with KSOM medium. The blastocysts were transfected into diluted lentivirus for 6 h and then cleaned for use. (4) GD2 pseudopregnant mice were anesthetized and subcutaneously injected with 2mg/kg meloxicam into the back opening to nonsurgical embryo transfer into the uterus.

1.13. Intrauterine administration of lentivirus

Pregnant mouse GD14 was anesthetized and injected with 2mg/kg meloxicam subcutaneously. The uterus was removed on both sides, and lentivirus was injected into each placenta with sterile 34G Hamilton needles. After being put back into the uterus, 1ml PBS containing 100mg/kg cefazoline was filled into the abdomen to prevent infection, and the abdomen was sutured and placed in an incubator until the mice recovered.

1.14. Statistical analysis

The mean ± *SEM* was used to present quantified data. Statistical analysis of data was completed with *SPSS 23.0* software. The statistical significance of the differences was obtained through the parametric test (*Student's* t-test or *ANOVA*) and non-parametric test (*Mann-Whitney U* test or *Kruskal-Wallis T* test). For *ANOVA*, *Bonferroni* or *Tamhane's T2* method was further used after homogeneity test of variance. $P < 0.05$ was considered statistically significant.
